# Common garden comparisons confirm inherited differences in sensitivity to climate change between forest tree species

**DOI:** 10.7717/peerj.6213

**Published:** 2019-01-15

**Authors:** Cuauhtémoc Sáenz-Romero, Antoine Kremer, László Nagy, Éva Újvári-Jármay, Alexis Ducousso, Anikó Kóczán-Horváth, Jon Kehlet Hansen, Csaba Mátyás

**Affiliations:** 1 Instituto de Investigaciones Agropecuarias y Forestales, Universidad Michoacana de San Nicolás de Hidalgo, Morelia, Michoacán, México; 2 Unité Mixte de Recherche Biodiversité Gènes & Communautés (UMR 1202 BIOGECO), Institut National de la Recherche Agronomique (INRA)—Université de Bordeaux, Cestas, Aquitania, France; 3 Forest Research Institute, National Agricultural Research and Innovation Centre (NARIC), Sárvár, Hungary; 4 Faculty of Forestry, Institute of Environmental and Earth Sciences, University of Sopron, Sopron, Hungary; 5 Department of Geosciences and Natural Resource Management, Section for Forest, Nature and Biomass, University of Copenhagen, Frederiksberg, Denmark

**Keywords:** *Quercus petraea*, *Picea abies*, *Pinus sylvestris*, Climatic change, Climatic transfer distance, Response function, Mixed model, Forest decline, Assisted migration, *Fagus sylvatica*

## Abstract

The natural distribution, habitat, growth and evolutionary history of tree species are strongly dependent on ecological and genetic processes in ecosystems subject to fluctuating climatic conditions, but there have been few experimental comparisons of sensitivity between species. We compared the responses of two broadleaved tree species (*Fagus sylvatica* and *Quercus petraea*) and two conifer tree species (*Pinus sylvestris* and *Picea abies*) to climatic transfers by fitting models containing the same climatic variables. We used published data from European provenance test networks to model the responses of individual populations nested within species. A mixed model approach was applied to develop a response function for tree height over climatic transfer distance, taking into account the climatic conditions at both the seed source and the test location. The two broadleaved species had flat climatic response curves, indicating high levels of plasticity in populations, facilitating adaptation to a broader range of environments, and conferring a high potential for resilience in the face of climatic change. By contrast, the two conifer species had response curves with more pronounced slopes, indicating a lower resilience to climate change. This finding may reflect stronger genetic clines in *P. sylvestris* and *P. abies,* which constrain their climate responses to narrower climatic ranges. The response functions had maxima that deviated from the expected maximum productivity in the climate of provenance towards cooler/moister climate conditions, which we interpreted as an adaptation lag. Unilateral, linear regression analyses following transfer to warmer and drier sites confirmed a decline in productivity, predictive of the likely impact of ongoing climate change on forest populations. The responses to mimicked climate change evaluated here are of considerable interest for forestry and ecology, supporting projections of expected performance based on “real-time” field data.

## Introduction

Analyses of quaternary and recent range shifts have indicated that tree species migrate at different rates, due to differences in their migration abilities and successional behavior, but probably also due to differences in their sensitivity to climate change; these differences in migration rate may have affected the composition of forest communities (e.g., Davis & Shaw, 2001; Lenoir et al., 2008). An understanding of the determinants of the response of forest trees to changing environmental conditions is of fundamental importance, as the stability and productivity of forest cover plays a vital role in mitigating the negative effects of climate change.

Many predictions have been made concerning the likely impact of climatic change on forest tree species (e.g., Zimmermann et al., 2009; Cheaib et al., 2012). However, there are gaps in our knowledge concerning the contribution of differences in sensitivity to climate change between species, taking into account the pattern of genetic differentiation between populations within species. Large-scale shifts in growing conditions triggered by rapid climate change lead to changes in growth rate and shifts in species habitats during the lifetime of tree populations. Field studies are therefore required to clarify the role of genetic differentiation in these processes. Common garden data (provenance tests) for forest tree species have shown that broadly distributed forest tree species form genetically specialized populations, each of which is assumed to be optimally or almost optimally adapted to only a portion of the climatic niche occupied by the species as a whole (Morgenstern, 1996; Rehfeldt et al., 2017; Sáenz-Romero et al., 2017). Given the general shortage of field-based data concerning genetic adaptation, common gardens may provide useful information (Mátyás, 1994; Mátyás, Nagy & Újvári-Jármay, 2010; Mátyás et al., 2010; Leites et al., 2012a; Leites et al., 2012b; Rehfeldt et al., 2017; Sáenz-Romero et al., 2017), even though their datasets are mostly imperfect, for biological, technical and conceptual reasons. These common gardens were set up for other purposes, principally for identifying the best provenances for plantation programs, as genecological perspectives and estimation of the impact of climate change had not yet become relevant at the time of planting. However, the transplantation of populations into common gardens may be interpreted as mimicking climatic change, and the impact of such change can be characterized by climatic transfer distance (ecodistance, *sensu* Mátyás, 1994). The term “ecodistance” is defined as the difference between the investigated, ecologically relevant variables (in this case, climate) at the test site and at the population provenance (origin). Here, we revisit forest tree provenance tests originally designed decades ago for purposes other than predicting the impact of climate change. These data provide a unique opportunity to measure the response of trees in relation to ecodistance. If we substitute climatic space for time, we can use these data to anticipate the effects of ongoing climatic change on trees.

In general, populations respond to shifts away from their “optimum” climate by a decrease in growth and survival that can be characterized by a quadratic function (Mátyás & Yeatman, 1992; Leites et al., 2012a; Rehfeldt et al., 2017; Sáenz-Romero et al., 2017). Many of the dominant tree species in temperate zones have been analyzed for within-species differentiation in common garden tests (see the classic reviews by Langlet, 1971; Morgenstern, 1996). However, up to now mainly conifer species of the Northern Hemisphere have been studied (Mátyás & Yeatman, 1992; Shutyaev & Giertych, 1998; Shutyaev & Giertych, 2000; Rehfeldt, Ying & Spittlehouse, 1999; Rehfeldt et al., 2003; Rehfeldt et al., 2014; Tchebakova, Rehfeldt & Parfenova, 2005; Wang et al., 2006; Wang, O’Neill & Aitken, 2010; Kapeller et al., 2012; Chakraborty et al., 2015; Újvári-Jármay, Nagy & Mátyás, 2016), and there have been far fewer studies of broadleaved tree species (e.g., Horváth & Mátyás, 2016; Frank et al., 2017a; Sáenz-Romero et al., 2017). In particular, we are currently lacking a comparison of the pattern of phenotypic response between species with different evolutionary profiles and life histories. Genomic approaches can disentangle the evolutionary and demographic processes underlying population differentiation within species, but they cannot yet provide insight into the phenotypic response of fitness-related traits, such as growth or survival. In this respect, common garden experiments remain of the utmost importance for investigating adaptive responses to climate variation.

In this study, we compared the phenotypic responses of four dominant forest tree species widely distributed in temperate Europe: two conifer tree species, *Pinus sylvestris* (Scots pine) and *Picea abies* (Norway spruce), and two broadleaved tree species, *Fagus sylvatica* (European beech) and *Quercus petraea* (sessile oak), with a view to identifying differences in their responses to different climates. These four species are of great ecological and economic relevance in European forests (Shutyaev & Giertych, 1998; Shutyaev & Giertych, 2000; Rehfeldt et al., 2003; Tchebakova, Rehfeldt & Parfenova, 2005; Kapeller et al., 2012; Horváth & Mátyás, 2016; Újvári-Jármay, Nagy & Mátyás, 2016; Frank et al., 2017a; Sáenz-Romero et al., 2017). Height at the late juvenile stage (8–17 years) was used to compare populations. Previous studies have indicated that data at this age provide the first useful estimates of expected performance at more mature ages (Lambeth, 1980). The data used for this study were extracted from publications relating to extensive field provenance tests in Europe. We used the same functions and the same climate variables for each species, to make comparisons possible, rather than the usual procedure of selecting the climatic factors best explaining the observed variance by the modeling of individual species.

The principal research questions addressed by this study were: (a) Do the growth responses of populations transferred to different climates differ between tree species? (b) If they do, is part of the differential response embedded in the genetic variation among the source populations? (c) If such differential responses to climate exist between species and populations, do they contribute to the observed variation in growth decline, mortality or local extinction as a result of climate change (Allen et al., 2010; Allen, Breshears & McDowell, 2015; Mátyás, 2010)?

## Material and Methods

### Sources of data

In the framework of the European Union joint project FORGER (#289119), the response functions (reaction norms) of these four model species were investigated in European provenance tests by the Hungarian and French authors of this study. Part of the dataset from these multiple field provenance tests was used for this analysis. Fourteen populations of Scots pine (*Pinus sylvestris)* were selected from four trials performed by the VNIILM Institute, Pushkino, Russia (Shutyaev & Giertych, 1998; Shutyaev & Giertych, 2000); 34 populations were chosen from five trials of the international Norway spruce (*Picea abies)* trial series of IUFRO (Újvári-Jármay, Nagy & Mátyás, 2016); nine populations of European beech (*Fagus sylvatica)* were selected from six trials of an international IUFRO series (Horváth & Mátyás, 2016) and 14 populations of sessile oak (*Quercus petraea)* from 23 trials of the “Madsen collection” were used (Madsen, 1990; Sáenz-Romero et al., 2017).

Both the test sites and populations selected for the comparison were highly diverse. Layouts and test ages were not uniform, because the initial experiments had different individual goals and constraints concerning seed procurement and the planting of material due to seed crop fluctuations and differences in site accessibility. Randomized complete block designs were used for all four species, with blocks and replicated plots (for *Picea abies,* replicated single-tree plots) to account for within-site variation. The selection of populations for analysis was based on their presence in the trials (the populations included in trials were not the same everywhere) and data availability.

**Figure 1:**
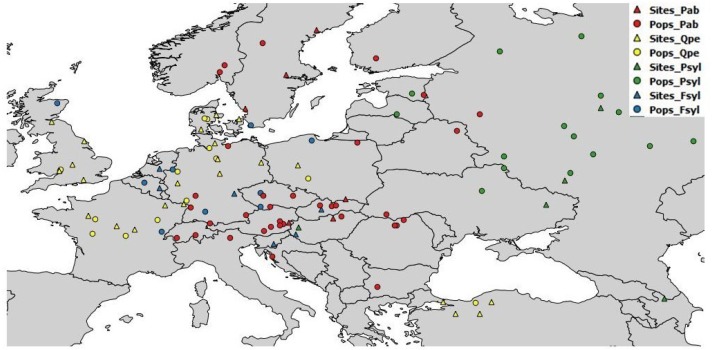
Geographic location of sampled populations and tests sites per species. Pab, *Picea abies*; Psyl, *Pinus sylvestris*; Fsyl, *Fagus sylvatica* and Qpe, *Quercus petraea*. Circles indicate provenances and triangles indicate field test sites.

The field tests were managed independently by the participating partners, according to their own management guidelines and standard silvicultural practices. Details of the test sites and populations used are provided in the Supplementary Information. Geographic and basic climatic data for the chosen populations are provided, by species, in Table S1; data for the test sites are provided in Table S2. A map of the seed sources and test sites is provided in Fig. 1. Age at tree measurement varied between the available databases. Height was measured at the age of eight years (from planting) for European beech and sessile oak, 12–17 years for Scots pine, and 16–19 years for Norway spruce (Table S3).

**Figure 2:**
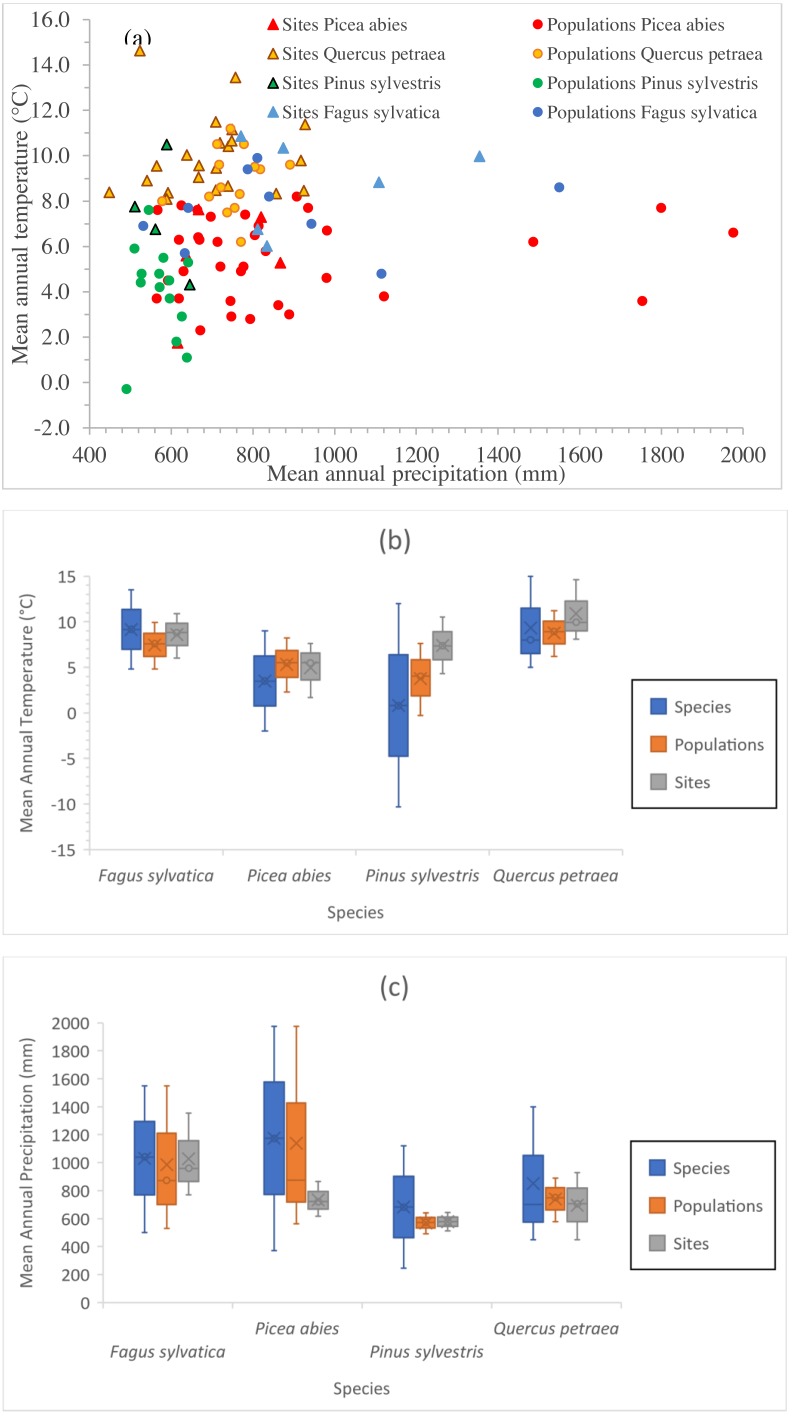
Climate space of species, populations sampled and tests sites. (A) Position of sampled populations and trial sites in the climatic space (not all locations are visible). Explanation of symbols as in Fig. 1. Climate interval covered by provenances represented in the field tests and by the tests sites for Mean Annual Temperature (B) and for Mean Annual Precipitation (C). For species interval, data were gathered from our own estimations (Tables S1 and S2), and from the following sources: *Fagus sylvatica*: Giertych & Mátyás (1991) and Fang & Lechowicz (2006); *Picea abies*: Schmidt-Vogt (1977); *Q. petraea* Truffaut et al. (2017); *Pinus sylvestris*: Rehfeldt et al. (2002) and Tchebakova, Rehfeldt & Parfenova (2010), personal communication with Nadja Tchebakova (V.N. Sukachev Institute of Forest, Siberian Branch, Russian Academy of Sciences; October 4th, 2018).

The positions of the sampled populations and trial sites in the climatic space are presented in Fig. 2A, and the climate ranges covered by the provenances represented in the field tests and of the test sites relative to the species climate range are shown in Figs. 2B and 2C. The four species have very different geographic distributions, but the sampled ranges overlap at least partially and sufficiently for comparison. The test sites were in average slightly warmer than the sites of origin of the populations tested. At the time at which the tests were established, well before climate change became a major concern, it was usual to establish such experiments in favorable site conditions. The shift in climate conditions already underway at the time may have contributed to this. (Note that the reference climate periods for provenance locations and test sites are different—see below.) The locations of the provenance and field test sites for *P. sylvestris* cover a much smaller interval than the species as a whole, due to the relatively limited size of the range selected for investigation relative to the immense natural distribution of this species.

For the statistical analysis, we used means per population per block, except for the *Picea abies* trial network, which was established as a single-tree plot system with 25 replicate, for which means per site and per population were used. For technical reasons, our dataset was not balanced across species, as different numbers of populations and field sites per species were included in the analysis.

Hereafter, we refer to the assemblage of mother trees (forest stands, compartments, from which seeds were collected), represented by their seedlings in the experiments, as “populations”, whereas the origin of the population (the sampling site) is referred to as their “provenance” or “seed source”.

### Climate data

Contemporary climate data for each provenance and test site were estimated from the data of Wang & Hamann (2012). Climate variables are described in Table 1. The time periods used as a reference are listed in Table S3. The climate estimates for the seed sources (provenance locations) and for the test sites were estimated for different time spans, as described by Horváth & Mátyás (2016). For the provenance climate, the earliest available climate data were used, to characterize the conditions to which populations had adapted in the past, through a long process of evolution. Climate data for 1961–1990 were used for the normalized climate period immediately before seed collection for *Fagus sylvatica* and *Quercus petraea*. These reference periods are the best estimates of the conditions responsible for the semblance of adaptive equilibrium before human-induced climate change began to accentuate (Sáenz-Romero et al., 2015). In the case of *Pinus sylvestris* and *Picea abies*, for which seeds were selected earlier than for the broadleaved species, we used the mean climate data estimates for the 1941–1970 period. The climate data estimates for the test sites were calculated as the mean values for the period from planting to measurement, as in Table S3 , to account for the effect of the climate ^1^ 1The growth periods in the trials were actually shorter than the standard 30 years usually required for the term “climate” to be used.in which the trees were actually growing.

**Table 1:** Climate variables used for the modeling. Code, definition and units of the climatic variables used, after Wang & Hamann (2012). Annual Dryness Index based on Rehfeldt (2006).

Code	Definition and units
*Annual variables. Directly calculated variables:*	
MAT	mean annual temperature (°C)
MWMT	mean warmest month temperature (°C)
MCMT	mean coldest month temperature (°C)
TD	temperature difference between MWMT and MCMT, or continentality (°C)
MAP	mean annual precipitation (mm)
MSP	mean summer (May to Sept.) precipitation (mm)
AH:M	annual heat/moisture index ((MAT +10)/(MAP/1,000))
SH:M	summer heat/moisture index ((MWMT)/(MSP/1,000))
*Annual variables. Derived variables*	
DD<0	degree-days below 0 °C, chilling degree-days
DD>5	degree-days above 5 °C, growing degree-days
DD<18	degree-days below 18 °C, cooling degree-days
DD>18	degree-days above 18 °C, heating degree-days
NFFD	the number of frost-free days
FFP	frost-free period
bFFP	the Julian date on which FFP begins
eFFP	the Julian date on which FFP ends
PAS	precipitation as snow (mm) between August in previous year and July in current year
EMT	extreme minimum temperature over 30 years
Eref	Hargreaves reference evaporation
CMD	Hargreaves climatic moisture deficit
ADI	Annual Dryness Index ((DD > 5)^1/2^/MAP)
*Seasonal variables*	
TAV_wt	winter (Dec.(prev. yr)–Feb.) mean temperature (°C)
TAV_sp	spring (Mar.–May) mean temperature (°C)
TAV_sm	summer (Jun.–Aug.) mean temperature (°C)
TAV_at	autumn (Sep.–Nov.) mean temperature (°C)
TMAX_wt	winter mean maximum temperature (°C)
TMAX_sp	spring mean maximum temperature (°C)
TMAX_sm	summer mean maximum temperature (°C)
TMAX_at	autumn mean maximum temperature (°C)
TMIN_wt	winter mean minimum temperature (°C)
TMIN_sp	spring mean minimum temperature (°C)
TMIN_sm	summer mean minimum temperature (°C)
TMIN_at	autumn mean minimum temperature (°C)
PPT_wt	winter precipitation (mm)
PPT_sp	spring precipitation (mm)
PPT_sm	summer precipitation (mm)
PPT_at	autumn precipitation (mm)

### Statistical analysis

The main challenge in the comparison of climate sensitivity between different species was identifying the climate variable giving the best fit to the response curve.

The climatic niches of different species are obviously not defined by the same climatic variables, as individual climatic variables can have different impacts on growth in different species. A compromise was therefore required to achieve an instructive comparison of response functions between the four forest tree species. For this purpose, we developed a common model, including the same climatic variables, for all four species. This may appear counterintuitive, but it should be borne in mind that the ecological tolerance range of a species is generally characterized with a single climate factor, even though the effect of this factor has different weights at the trailing (xeric) and front (thermal) limits of the distribution. We adopted a two-step approach to select the best climatic variables. We first explored the overall fit to a global model including the species and population components, by screening a large number of climatic variables. Once the most decisive climatic variables had been identified, we then performed a separate analysis for each species, to retrieve the species reaction norms, which we then compared.

### Defining the response to transfer between climates: application of a mixed model

We checked the consistency of the results, using tree height as the dependent variable. We then used tree height and annual tree height increment as dependent variables, for modeling of the plant response to climate at species level, analyzing genetic differences between populations nested within species, with the approach described by Leites et al. (2012a) and Leites et al. (2012b). The mixed effects model (SAS/STAT computer software, release 9.1; SAS Institute Inc., Cary, NC, USA) includes both fixed and random effects. The fixed effects in this context correspond to genetic differences between populations due to climatic conditions, and can be split into three major components (Rehfeldt et al., 2003; Leites et al., 2012a; Leites et al., 2012b; Sáenz-Romero et al., 2017): (a) The effect of climate at the site of provenance, term “*C*” of the model, which accounts for genetic differentiation between populations due to climate at the provenance site, as a result of past climatic selection. (b) The climatic transfer distance, term “*D*” [*D* = (climate at test site) − (climate at provenance); linear or quadratic], which accounts for population differentiation due to differences in climate between the provenance and test sites (Mátyás, 1994); this term can be used to characterize the sensitivity or plasticity of populations as a response to climate transfer, based on the shape of the response function (reaction norm) curve. (c) The *D*∗*C* term, which accounts for the interaction between climatic transfer distance and the climate of the provenance site; in other words, the response of a population to transfer distance may depend on the climate of origin.

The random effects of the model account for the sources of variation not explained by the fixed effects: of test sites (environmental effects other than climatic effects, such as soil fertility, texture, drainage, silvicultural maintenance regimes, etc.), of populations (genetic effects other than those due to selection by climate, such as genetic drift, gene flow or inbreeding), and of blocks (Leites et al., 2012a; Leites et al., 2012b).

The full model is very similar to that used and described in detail by Sáenz-Romero et al. (2017), except that we have added a species term and nested population within the species term. Our model can be expressed as follows:

(1) \begin{eqnarray*}& & {Y}_{ijkl}={\beta }_{0}+{\beta }_{1}{D}_{ijk}+{\beta }_{2}{D}_{ijk}^{2}+{\beta }_{3}{C}_{ik}+{\beta }_{4}({D}_{ijk}\ast {C}_{ik})+{S}_{i}+{T}_{j}+{P}_{k}({S}_{i})+{e}_{ijkl}\end{eqnarray*}

where *Y*_*ijk*_ is measured tree height (mean per block per population per site), of the *k*th population, nested in the *i*th species in the the *l*th block, nested in the *j*th test site; *β*_0_ is the intercept; *D*_*ijk*_ is the climatic transfer distance for the *k*th population nested in the *i*th species at the *j*th test site; *C*_*ik*_ is the value of the climate variable at the provenance of the *k*th population nested in the *i*th species; *D*_*ijk*_∗*C*_*ik*_ is the interaction between climatic transfer distance (for the *k*th population at the *j*th field test), and the climate variable at the provenance (of the *k*th population nested in the *i*th species); *β*_1_–*β*_4_ are regression coefficients; *S*_*i*_ is a random species effect of the *i*th species; *T*_*j*_ is a random site effect for the *j*th test. *P*_*k*_(*S*_*i*_) is a random population effect of the *k*th population nested in the *i*th species, due to factors other than climate; *e*_*ijkl*_ is the error term. *D*_*ijk*_, *C*_*ik*_ and *D*_*ijk*_∗*C*_*ik*_ are fixed effects. *S*_*i*_, *T*_*j*_ and *P*_*k*_ are random effects (Leites et al., 2012a; Leites et al., 2012b).

This model was adopted on the basis of the ecological assumption that the response function (reaction norm) of populations to any change of climate follows a quadratic, symmetric “bell-shaped” function with a maximum at, or near a transfer distance of 0, i.e., close to the climate of the provenance, assuming local adaptation (Wang, O’Neill & Aitken, 2010; Kapeller et al., 2012; Leites et al., 2012a; Leites et al., 2012b; Yang et al., 2015; Sáenz-Romero et al., 2017). The response to climatic transfer includes a quadratic function (*D*^2^ term), but the term for climate at seed source (*C* term) is based on a linear function. A large body of experimental results from common gardens of forest trees is available, indicating a linear, clinal variation of populations along climatic or geographic gradients (Mátyás & Yeatman, 1992; Alberto et al., 2013; for a review: Morgenstern, 1996).

A species-specific model was also applied. This model is similar to that described above, but without the species term:

(2) \begin{eqnarray*}& & {Y}_{jkl}={\beta }_{0}+{\beta }_{1}{D}_{jk}+{\beta }_{2}{D}_{jk}^{2}+{\beta }_{3}{C}_{k}+{\beta }_{4}({D}_{jk}\ast {C}_{jk})+{T}_{j}+{P}_{k}+{e}_{jkl}.\end{eqnarray*}

We used a screening procedure to select climate variables for the linear and quadratic functions (Appendix S2). We found a single provenance climate variable appropriately expressing the clinal variation between populations. The inclusion of additional variables reduced the statistical quality of the models. In theory, multivariate climate transfer effects are intuitively appealing, but they increase statistical complexity and may lead to insurmountable constraints.

### Fitting of an “overall” (full) model and species-specific models

We first fitted an “overall” transfer function model, with all four species, and populations nested within species (model 1), to identify the best mixed model with common climatic variables (one for provenance, one for climatic transfer distance). The best variable combination for the response functions (reaction norms) was determined simultaneously for the four species. We compared 49 models, and the one with the lowest AIC (Akaike Information Criterion, Akaike, 1973) value was selected as the best model. Model selection, including preselection of the seven best models for provenance and the seven best models for climatic transfer distance, yielding a matrix of 49 competing models, is described in Appendix S2.

We then refitted the model for each species separately, with the same selected variables, but using only observed data for the species concerned as input, to generate appropriate parameter values for the fixed-effect terms, and individual response functions for each species. These response functions were illustrated by plotting a curve for each species, using the model parameters estimated for the fixed terms for each species, and plotting the mean value of the selected climate variable for the species at the seed source against the best climatic transfer distance variable. The range of climatic transfer distance values (*x* axis) was extended beyond the measured values to make it possible to visualize the response function trend in full, as most of the field trials were not located at sites with extreme climates.

We checked that the differences in response curve between species persisted even if a different set of climate variables was used, by fitting response curves independently for each species. In this validation process, we selected the best climatic variable for each species separately with model 2. We thus obtained four pairs of climatic variables (for the *D* and *C* terms for each species). We then used each pair of variables obtained for a given species on the other three species, implementing model 2. This procedure yielded 16 models in total.

For further improvement of the comparison between species, we repeated the process for full model fitting with the selected climatic variables from models 1 and 2 above, this time using scaled growth data as an independent variable, to lessen differences in age and in species growth potential. We applied the following formula for each subset of data (for each age nested within each species):

\begin{eqnarray*}& & \mathrm{scaled~ height}=[\mathrm{height}-(\mathrm{minimum~ height})]/(\mathrm{maximum~ height}-\mathrm{minimum~ height}). \end{eqnarray*}

We then used the estimated fixed-term parameters to determine scaled-height response functions for each species, using the common climatic variables. In this analysis, “tree height” is the mean tree height per block.

We repeated the three-step model selection process for annual tree height increment (ATHI = tree height/age) and plotted the predicted values for each species against climatic transfer distance, to confirm the previous results and to eliminate age differences between species.

### Exploring genetic clines

We explored the amount of genetic differentiation between populations, probably linked to the shape of the response function, by investigating the expression of clines along environmental gradients, by screening relevant provenance climate variables. We estimated Spearman’s correlation coefficients for the relationship between the mean ATHI per population per site and the provenance climate variables. We analyzed only “good sites” (sites at which the mean per population per site was above the overall mean), as differences in growth potential between populations are best expressed in mild environmental conditions. We ranked the absolute values of Spearman’s correlation coefficient by species. We then selected the three climate variables with the highest Spearman correlation coefficient values for each species. The site effect was eliminated by estimating the best linear unbiased parameter (BLUP) value for each site, and subtracting it from the provenance mean per site. BLUPs were estimated with a reduced mixed model, in which climate at provenance was a fixed effect and site was a random effect. The next step was to standardize the described corrected mean per population per site, by dividing it by the overall mean (across provenances and sites). In other words, we obtained standardized corrected means for each population and site, with a distribution centered on zero. Finally, we performed a linear regression analysis of the standardized corrected means per population per site against the provenance climate values of the three most relevant climatic variables per species identified in previous analyses, to compare the fit and slope of the regression lines. We plotted the regressions, to obtain a visual comparison of the different cline patterns between species.

### Fitting a model for unilateral climatic transfer, towards warming

The global climate is changing in a single direction, with a marked shift towards warming. When modeling the future responses of species and populations to climate change, it is important to remember that only efforts to mimic such unilateral change, based on the transfer of populations to warmer and drier conditions, are of practical relevance.

We performed a regression analysis for unilateral climate transfer, with a shift towards warmer/drier climatic conditions, using the same climatic variables in the chosen model. In other words, we regressed the means per population per site against positive climatic transfer distance values only. In biological terms, a second-degree regression model would be plausible, but we nevertheless fitted a simple linear regression model, because the data were highly scattered and we wished to avoid artifactual overfitting. The linear model used for each species was:

(3) \begin{eqnarray*}& & {Y}_{jkl}={\beta }_{0}+{\beta }_{1}{D}_{jk}+{e}_{jkl}\end{eqnarray*}

where *D*_*jk*_ is the climatic transfer distance for the *k*th population, of a given species, at the the *j*th test site.

## Results

### Fitting of the “overall” (full) model: determining the best variables for the climate transfer function

After preselecting the climatic variables and identifying the overall (full) model best explaining the variability (for all four species), we applied a transfer distance based on annual dryness index (ADI) as the *D* term of the mixed model and used winter mean maximum temperature (°C) of the climate at provenance as the *C* term of the mixed model. The statistics of the model with the selected variables are presented in Table 2. Details of model selection and a comparison of fits with models based on other climatic variables can be found in Appendix S2 and Table S4.

**Table 2:** Statistics of the mixed model analysis of tree height for the “overall” model (all the four species). Akaike Information Criterion (AIC), estimated parameters, contribution to total variance of random components and significance.

Parameter/components of variation of tree height	Statistics		
Fixed effects	Estimated parameter or variance value	%^a^	*P*
AIC	22089.6		
Intercept	394.6		0.0289
Winter mean maximum temperature at provenance (TMAX_wt_s)	0.57		0.8279
Annual dryness index transfer distance (ADI_d)	−1275.8		0.0048
Annual dryness index transfer distance quadratic (ADI_d)^2^	−9635.3		0.0937
Winter mean maximum temperature at seed source x annual dryness index transfer distance	45.1		0.3013
*Random effects*			
*Species*	36,848	63.0	0.1315
*Site*	17,198	29.4	<0.0001
*Population (Species)*	1,953	3.3	<0.0001
*Error*	2,464	4.2	<0.0001

**Notes.**

a Percent contribution to total variance (where 100% is the sum of variance of all random terms).

An examination of the virtual response surface emerging from the results showed that linear and quadratic values of term *D* controlled the response of populations (i.e., height growth was determined principally by the effect of climate transfer), with term *C*, winter mean maximum temperature at provenance playing only a minor, secondary role.

**Figure 3:**
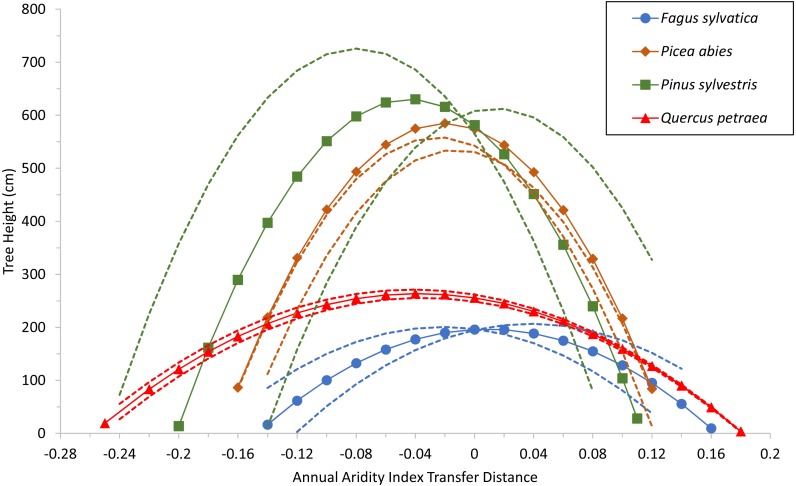
Tree growth response to climatic transfer distance. Predicted tree height (cm) of species averages (solid lines and symbols) and of extreme populations per species (with coldest or warmest winter temperatures; broken lines) vs. climatic transfer distance, for *Fagus sylvatica, Quercus petraea*, *Pinus sylvestris* and *Picea abies*. Positive values on the *x* axis indicate transfer to drier and/or warmer sites; negative values signify transfer to cooler and/or moister sites; zero stands for a climate similar to that at the site of provenance (further explanation in text).

Specific values for the two common variables selected (corresponding to terms *C* and *D*) were used for each species as well, to build a model with estimated parameters of the fixed-effect terms for each species. The quadratic response functions are shown for the species in Fig. 3. Curve height is related to the age of the populations at the time of measurement (see Table S3). The curves for the two conifers are therefore higher than those for the broadleaved trees because the trials were older. Thus, when comparing the curves, width and steepness should be considered, rather than maxima.

The model shows that growth decreases when populations are transferred either to warmer/drier sites (positive transfer distances), or to cooler/wetter sites (negative values). The shape of the response curves in Fig. 3 is of particular importance: flat and wide for *Fagus sylvatica* and *Quercus petraea*, but with more pronounced slopes for *Pinus sylvestris* and *Picea abies*. This means that the (adaptive) responses of populations to change are much more marked for the two conifer species. This results from the ADI^2^ quadratic fixed term, which determines the shape of the response curves and highlights the differences between the modeled conifers *Pinus sylvestris* and *Picea abies*, and the modeled broadleaved species *Fagus sylvatica* and *Quercus petraea*.

### Species transfer response

The trend shown in Fig. 3 was confirmed by the fitting of separate response curves for each species, with the three-step model selection procedure repeated for each species. The shapes of the curves obtained were generally similar to those in Fig. 3, except that the quadratic term yielded a positive value in some cases, resulting in an inverted, biologically unacceptable curve, which was therefore discarded. Curves of this type fitted for *Fagus sylvatica* (Fig. S1A) and for *Pinus sylvestris* (Fig. S1B) are presented in the supplementary information.

The response functions (reaction norms) did not have maxima close to a transfer distance of zero (i.e., corresponding to climates identical to the climate of provenance) for any species other than *Fagus sylvatica*, for which the maximum was very close to zero (on the mean curve for this species, the maximum tree height of 196.6 cm corresponds to an annual aridity index transfer distance value of 0.01; Fig. 3). For *Picea abies* and *Quercus petraea*, the maximum height was slightly displaced towards cooler and/or moister sites (for the mean curve of these two species, maximum tree height was predicted to occur at an annual aridity index transfer distance value of −0.02 and −0.04, respectively; Fig. 3). A similar phenomenon was observed for *Pinus sylvestris* (maximum tree height at an annual aridity index transfer distance value of −0.045; Fig. 3). These results are consistent with the species-specific results of analyses of field tests (e.g., Mátyás, Nagy & Újvári-Jármay, 2010; Rehfeldt et al., 2017).

**Figure 4:**
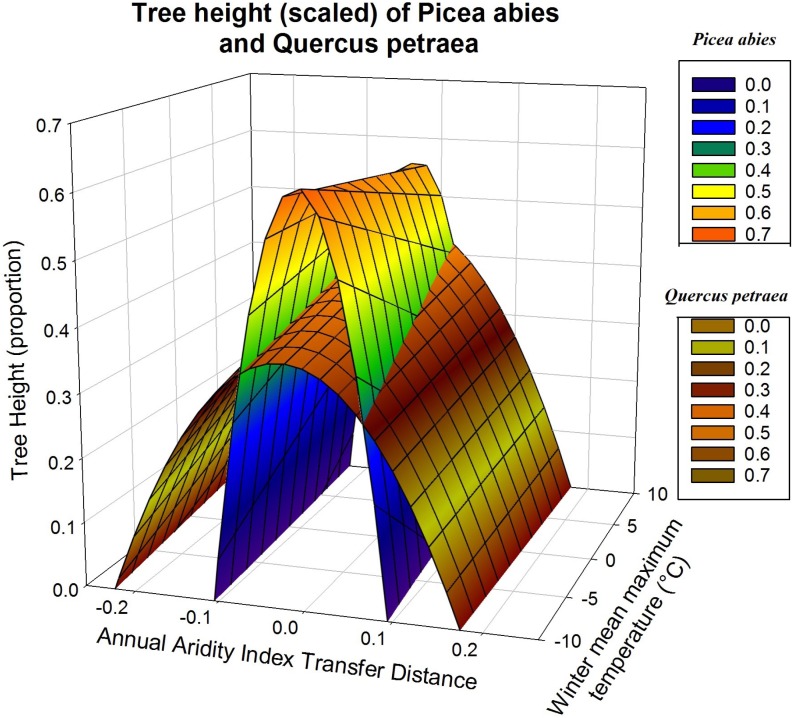
Tree growth response to climatic transfer distance with scaled data. Illustration of differences of response to climatic transfer on the example of predicted response surfaces of scaled tree heights for *Picea abies* (blue to orange data surface) and *Quercus petraea* (brownish data surface).

Response functions may also be displayed as a three-dimensional surface, with the two climatic variables determining measured height (Fig. 4). This illustrates more clearly the difference between the responses of *Q. petraea* and *P. abies*. *Q. petraea* has a flat response, encompassing a larger climatic interval under the response surface (a range of 0.41 for annual aridity index transfer distance, from about −0.23 to 0.18), whereas *Picea abies* has a much steeper and narrower surface, encompassing a smaller climatic space interval (a range of 0.20 for annual aridity index transfer distance, from about −0.11 to 0.09; Fig. 4).

As in Fig. 3, the predictions shown in Fig. 4 are based on the climatic variables selected for simultaneous fitting to all four species, with subsequent refitting of the model for each species, for the estimation of regression coefficients for each species, with scaled heights (only two of the four species are displayed for the sake of clarity). The quadratic *D* term and the linear term *C* determine the shape of the response curve. *C* covers the climatic gradient along which population samples were collected, whereas *D* describes the response to climatic transfer distance.

We also modeled the annual tree height increment (ATHI) response (by three-step model selection, Appendix S2, as for tree height) against annual dryness index, which was identified as the best variable to represent climatic transfer distance and temperature difference (TD = MTWM − MTCM = continentality), which was selected as the best variable to represent seed source climate. The results displayed in the supplementary information (Fig. S2; Table S6) reveal a trend similar to that for tree height (Fig. 3), confirming the general pattern of flatter response curves for *Q. petraea* and *F. sylvatica* and more pronounced, narrow curves for the two conifer species (Fig. S2).

### Genetic clines

**Figure 5:**
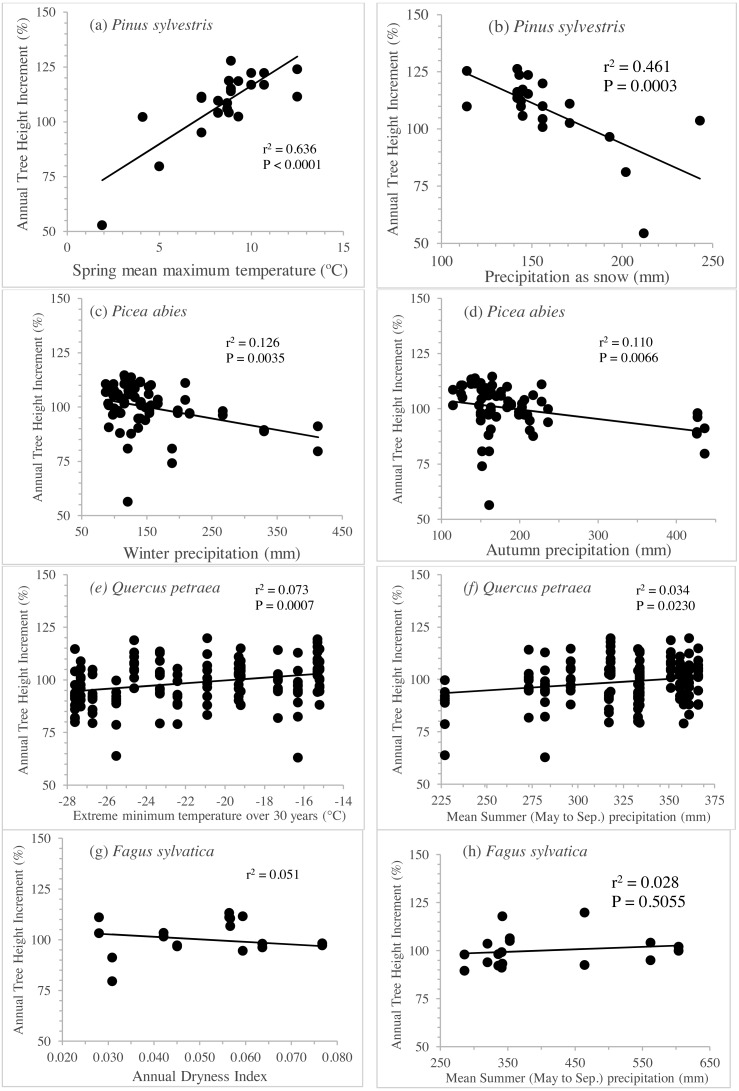
Clines per specie to illustrate genetic differentiation among populations. Annual tree height increment (previously corrected by subtracting the site effect and standardized), regressed against the best three seed source climate variables per species, for *Pinus sylvestris* (A, B), *Picea abies* (C, D), *Quercus petraea* (E, F) and *Fagus sylvatica* (G, H). Estimations based only on “good” tests sites (with annual tree height increment average per site above the overall average).

The separate exploration of clines for annual height increment within in each species revealed striking differences between species. Linear genetic clines were much more pronounced in *Pinus sylvestris*, along gradients of spring temperature or precipitation as snow (Figs. 5A and 5B), accounting for 43% to 64% of the total variance (Table 3). *Picea abies* displayed less pronounced but nevertheless highly significant clines (see Figs. 5C and 5D; 11% to 13% of the variance explained, *P* <0.007; Table 3) along gradients of winter or annual precipitation. By contrast, the clines for *Q. petraea* were weaker (Figs. 5D and 5E), explaining less than 8% of the variance (Table 3), and those for *Fagus sylvatica* (Figs. 5F and 5G) were weaker still, accounting for less than 6% of the variance (Table 3).

### Fitting a model for unilateral climatic transfer, with a shift towards warming

**Figure 6:**
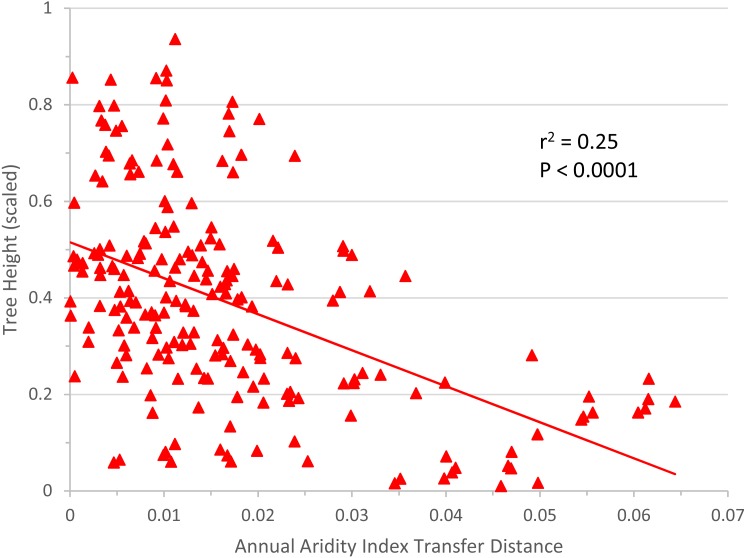
Response to transfer towards drier/warmer sites. Unilateral linear regression of average tree height (scaled) for all populations and all sites vs. annual aridity index transfer distance for *Q. petraea*. Only data towards the warmer/drier site conditions, i.e., with transfer distance ≥ 0 were considered.

The linear regression of annual aridity index transfer distance (independent variable) against mean scaled tree height per population (response variable) clearly showed, for *Q. petraea,* that growth decreases following transfer to warmer/drier sites (Fig. 6). The regression was highly significant for *Q. petraea* (*P* <0.001), and non-significant for *P. abies* and *Fagus sylvatica,* with *Pinus sylvestris* having an intermediate *P* value (0.070)*,* with growth tending to decrease with increasing climatic transfer distance towards warmer/drier sites. In this analysis, response functions were not extrapolated beyond the measured points, and data for negative transfer distance values were not considered. The high degree of scatter is largely due to the high within-species variance of individual populations.

**Table 3:** Clines per species. Slope, proportion of variance explained (%) and significance (*P*) for annual tree height increment (previously corrected by site effect and standardized), regressed against the best three seed source climate variables per species. Estimations based only on “good” tests sites (with annual tree height increment average per site above the overall average).

Species	Climate at seed source	Slope	%	*P*
*Pinus sylvestris*	Spring mean maximum temperature	63.2	63.6	<.0001
	Precipitation as snow	164.9	46.1	0.0003
	Annual heat/moisture index	42.3	43.3	0.0005
*Picea abies*	Winter precipitation	107.9	12.6	0.0035
	Autumn precipitation	108.2	11.0	0.0066
	Precipitation as snow	106.3	10.9	0.0068
*Quercus petraea*	Extreme minimum temperature over 30 years	113.1	7.3	0.0007
	Mean Summer (May–Sep.) precipitation	81.0	3.4	0.0230
	Summer precipitation	89.5	1.8	0.0969
*Fagus sylvatica*	Annual Dryness Index	106.4	5.1	0.3690
	Mean Summer (May–Sep.) precipitation	94.9	2.8	0.5055
	Summer precipitation	94.5	2.9	0.4963

## Discussion

Three major results emerge from this comparative analysis of species responses to climate change. First, the response patterns of *Pinus sylvestris* and *Picea abies* clearly contrast with those of *Fagus sylvatica* and *Quercus petraea* (e.g., the response curve of *Picea abies* encompasses about half the climatic transfer distance range covered by that of *Q. petraea*, as explained for Fig. 4). Second, these contrasting patterns (*P. sylvestris* and *P. abies* having more pronounced slopes than *F. sylvatica* and *Q. petraea*) coincide with the different genetic clines along climatic gradients established historically as a result of divergent selection across the species’ distributions, as illustrated in Fig. 5. Finally, populations of all four species tend to occupy suboptimal, stressful environments, i.e., they are present in climatic niches that are drier or warmer than the optimum for their growth.

### Contrasting response curves and genetic clines

Response curves based on climatic variables identified in provenance tests have already been investigated separately for different species (e.g., Rehfeldt et al., 2002; Mátyás & Nagy, 2005 for *P. sylvestris*; Horváth & Mátyás, 2016 for *Fagus sylvatica*; Sáenz-Romero et al., 2017 for *Q. petraea*), but we used a novel comparative approach in this study. Interestingly, our results are consistent with those of the previous studies, in which drought-indicating variables, not unlike annual dryness index (e.g., Ellenberg drought index, Horváth & Mátyás, 2016; summer drought period length, Frank et al., 2017a) were found to be significant.

The flatter climatic response curves of *Fagus sylvatica* and *Quercus petraea* (resulting in modest growth over a wider range of aridity indices than for *Pinus sylvestris* and *Picea abies*) indicates that the populations of these species have higher levels of plasticity, enabling them to adapt to a broader range of environments. The response curves of these species were also flat towards the cooler and more humid environments. By contrast, *Pinus sylvestris* and *Picea abies* have steeper response curves with more pronounced slopes, encompassing a narrower range of environmental conditions to which they appear to be adapted. These response curves suggest that spruce and pine are more vulnerable to climate change, as the pronounced cline slopes indicate a higher degree of specialization. In a context of assisted migration, the conifer response curves suggest a large advantage of re-aligning genotypes to the environment to which they are adapted. Transferring seed sources to sites at which the future climate is predicted to be similar to their current “home” climate is being treated as a possible option in operational forestry.

This conclusion is supported by European forest mortality observations. Drought-triggered mortality is observed regionally in oak and beech (e.g., Lakatos & Molnár, 2009), but the destructive decline of Norway spruce due to climate shifts is continent-wide and cannot be explained solely by the known artificial extension of its distribution range by humans (Seidl, Schelhaas & Lexer, 2011; Marini et al., 2017). A Europe-wide investigation of genetic conservation units for the most important forest tree species (Schueler et al., 2014) also revealed that the two conifer species studied here are exposed to significantly stronger climatic threats than oak and beech. Several studies have reported high sensitivity to drought in *P. abies* (Bošela et al., 2014; Bouriaud & Popa, 2009; Desplanque, Rolland & Schweingruber, 1999; Huang et al., 2017; Lebourgeois, Rathgeber & Ulrich, 2010; Lévesque et al., 2014; Zang et al., 2014).

The lower adaptability of *Pinus sylvestris* and *Picea abies* to climatic transfer distance may result from their pronounced differentiation into genetic clines along climatic gradients, due to the divergent selection that has historically occurred along these gradients. In other words, steeper genetic clines probably constrain the response of pine and spruce populations to narrower reaction norms. By contrast, *Fagus sylvatica* and *Quercus petraea* have milder clines, which contribute to flatter reaction norms. We suspect that the widths of the climatic gradients to which populations were exposed were relatively similar for the four species, as they occupy similar geographic ranges, at least in Central Europe. Differences in cline steepness between species are therefore likely to reflect intrinsic genetic differences between species.

Mechanisms other than adaptation to climate-imposed selection may also play an important role in the greater phenotypic plasticity of *Fagus sylvatica* and *Quercus petraea*. For example, these species may have more efficient mechanisms for dealing with biotic interactions, such as competition, antagonism, mutualism, defenses against insect and pest attacks, and a greater capacity to recover from such attacks. This hypothesis appears plausible in light of the recent decimation of natural conifer populations by beetle infestations linked to ongoing climate change (Allen et al., 2010; Alfaro et al., 2014).

It is tempting to conclude that the more plastic responses of *Fagus sylvatica* and *Quercus petraea* might be due to the shedding of their leaves in the fall, or in periods of severe drought, enabling them to escape the worst climatic stresses, but this is probably an oversimplification. Evolutionary genetic and ecophysiological background, and, thus, adaptive strategy, appear to differ between species, making simple generalizations difficult. Even within closely related species, such as those of the genus *Abies*, the between-species differences in plasticity measured in common gardens are considerable (Frydl et al., 2018). The plasticity of adaptive specialists, with a high between-population variance for adaptive traits, may result in higher levels of sensitivity to climatic conditions, as in Norway spruce. By contrast, in beech, which had a flat response curve to climate changes in our analysis, high levels of within-population genetic variation were detected in comparison tests on seedlings (Frank et al., 2017a; Frank et al., 2017b).

### Suboptimal presence of the four species

Our results indicate that the studied species (except for the adaptive generalist species beech, for which results were inconclusive) are preferentially present in environments that are drier or warmer than their optimum for growth. “Non-optimality” is a specific phenomenon of deviation from the expected symmetric shape of response curves. Asymmetric responses have been detected in common garden datasets (Mátyás & Yeatman, 1992; Rehfeldt et al., 2017). Early observations (Namkoong, 1969; Mátyás, 1990) indicated that response functions (reaction norms) constructed from common garden growth data displayed a systematic divergence of maxima from the mimicked position of climate similar to that of the provenance. This lag effect probably indicates a balance in the processes leading to local adaptation between directed selection and other random genetic effects, such as gene flow (see Savolainen, Pyhäjärvi & Knürr, 2007; Kremer et al., 2012). In conjunction with phenotypic plasticity, it may prevent “perfect” optimization, which would, in itself, lead to lower levels of survival in fluctuating environments (Mátyás, Nagy & Újvári-Jármay, 2010). Other factors (such as interspecific competition or other biotic interactions) may also have a strong effect on the contemporary distributions of natural populations.

We further extended “non optimality” towards drier and warmer sites, by analyzing responses to unilateral transfer. We thus focused on the most important practical question in forestry today: how the growth of populations is affected by a simulated shift in climate towards warmer/drier conditions. The simple linear regressions applied are free from extrapolation bias and confirmed the rapid loss of productivity under the anticipated changes in climatic conditions, but this trend has already been reported in species-specific analyses of common garden tests (Sáenz-Romero et al., 2017; Mátyás, Nagy & Újvári-Jármay, 2010; Újvári-Jármay, Nagy & Mátyás, 2016; Horváth & Mátyás, 2016). These results highlight the need to develop better models for the lower (xeric) limits, which has become particularly urgent in light of predicted climate change (Mátyás, 2010).

### Limitations of this study

We acknowledge that our conclusions regarding the contrasting responses of species may be driven by constraints inherent to the comparative approach used. The selection of a single “universal” climate variable, such as ADI (annual dryness index) seems, intuitively, to hinder the combined analysis of four species with different ecological niches. This approach undoubtedly increased the proportion of the variance remaining unexplained and lowered the significance of the results. However, given the continent-wide shift in climatic conditions at all sites, all tree species are simultaneously confronted with a real threat to their vitality and a risk of mortality across the distribution area: extreme droughts (Allen et al., 2010; Allen, Breshears & McDowell, 2015). ADI transfer difference (ADI_d) expresses the balance between temperature (degree days above 5 °C) and water availability, and, indirectly, the probability, intensity and frequency of extreme events regardless of geographic location. Through the use of differential values (transfer distances), we were able to take the *relative* differentiation due to climatic change across the geographic range into account.

Another constraint inherent to the comparative approach relates to that the choice of model. We assumed a quadratic response for each species, based on the generally accepted ecological assumption that the response of populations to changes in environmental factors follows a symmetric “bell-shaped” curve (e.g., Griffiths et al., 2000). The quadratic response model has proved successful for explaining variation in boreal conifers, but appears to be less effective for other species, including broadleaved species in particular, which display greater phenotypic plasticity in their response to site attributes, including climate.

The calculated parameter statistics for the mixed model confirm that the species-specific growth responses of populations are confounded with tree age, and partially with local site conditions. The largest variance component was the species effect (63.0% of random effects, see Table 1), but this value explains above all differences in age and site potential. The contribution of the exclusively genetic between-population component appears to be modest (3.3%, Table 1). It should be stressed that this figure accounts for only a part of the genetic variation captured by the analysis. Three fixed components (*D*, *D*^2^ and *C*; related to climate as a selective force), and the random component (resulting from effects other than climate) also contribute to genetic variation within species, and the transfer distance explains provenance-by-environment interactions.

The unbalanced, heterogeneous experimental setting may also have affected the outcome of this study. For researchers not familiar with field data from continent-wide common garden test series, the low level of model fitting across species may appear too uncertain for any practical conclusions to be drawn. A number of uncontrollable factors may have biased the response of individual species relative to others. In addition to management practices, important interacting biotic factors (e.g., differential pest pressures), and variations of local site conditions (fertility and water-holding capacity of the soil, hydrology) may be confounded with climatic effects. The consideration of site potential, as understood in forestry (an inseparable complex of soil, water and climate factors) might have accounted for much of the remaining variation. Insufficient detail in the description of experimental sites contributed to the high level of unexplained variation. In an international experimental series, in which data retrieval is dependent on different providers in different countries, it can be very challenging to obtain meaningful site data (exposure, soil type, rainfall, microclimate etc.) because of missing data for standard descriptors. Even the assessment of the most important abiotic factor in this analysis, climate, is dependent on the use of statistical concepts to interpolate digital surfaces from data points corresponding to meteorological observations. This situation, which is typical for large-scale common garden tests, highlights the need to describe site characteristics more accurately, to increase the predictive power of these valuable and often unique experiments.

Finally, the climatic range encompassed by the sampled populations and test sites for *Pinus sylvestris* and by the test sites for *Picea abies* (particularly for rainfall for *Picea abies*), being smaller than covered by the natural distributions of these species, may have limited our capacity to model the response function for the species as a whole with sufficient robustness. However, even with this limitation, we were able to capture the pattern of genetic differentiation between populations, with the detection of very pronounced clines for *P. sylvestris,* as shown in Fig. 5.

Despite these inherent limitations, our results were consistent across the various empirical validation steps applied, such as the use of different environmental variables, or raw vs. scaled data.

## Conclusions

The analysis of responses to climatic transfer showed that the height growth response was determined primarily by the extent of change (i.e., by the ecodistance of climate transfer), whereas climate conditions at provenance, the site to which the populations were originally adapted, played a more limited role. This study is original in its demonstration of response functions of different shapes in the four species studied. The two broadleaved species had wide flat response curves, whereas the two conifers had steep and narrow curves of response to changes in climatic factors. This differential response can be interpreted as evidence for differences in climate sensitivity between the two pairs of species.

Sessile oak and European beech appear to have higher levels of phenotypic plasticity, enabling them to adapt to a broader range of environments and, consequently, to display greater resilience in response to climate change. The response functions showed Norway spruce and Scots pine to be more sensitive to climate (less adaptable), which appears to be consistent with current observations of a greater climatic threat to conifers, particularly Norway spruce in Europe (Seidl, Schelhaas & Lexer, 2011; Marini et al., 2017; Schueler et al., 2014).

The steeper genetic differentiation clines in populations of *Pinus sylvestris* and *Picea abies* probably contribute to their sharper response to climate, as demonstrated by their transfer functions. By contrast, *Fagus sylvatica* and *Quercus petraea* had shallower genetic clines, probably accounting for their flatter climate response curves.

The maxima of the response functions were displaced towards cooler/moister climate conditions, deviating from the expected maximum productivity in the climate of provenance, for all species except beech. This finding was interpreted to reflect an adaptation lag and probably indicates that processes of local adaptation include other genetic effects and phenotypic plasticity, in addition to selection, preventing “genetically perfect” optimization.

The “real-time” phenotypic performance in response to climate change studied here is of greater practical value in forestry. By mimicking the response to shifts in climate factors, we can glimpse into the future, and project likely performance in field conditions.

##  Supplemental Information

10.7717/peerj.6213/supp-1 Table S1 List and basic data of populations tested Codes and **definitions of** climate variables as in Table 1.

10.7717/peerj.6213/supp-2 Table S2 Test sites per species See Table 1 for codes **and definition** of climate variables.

10.7717/peerj.6213/supp-3 Table S3 Time periods for estimated climate of seed sources and for climate at test sites Age of measurements considered are indicated.

10.7717/peerj.6213/supp-4 Table S4 Selection of climatic variables and best overall model for tree height Preselected best seven transfer distance climatic variables for tree height (Step 1). Selection was based on the AIC and the significance (*P*) of the transfer distance climatic quadratic term. Preselected seed source climatic variables (Step 2). Selection was based on Spearman’s rank correlation coefficient (*r*), between population means across sites and the seed source climatic variable. The best full model (Step 3) were selected on the basis of AIC value (after fitting the model with individual values). In each step, models are sorted by their AIC or absolute Spearman’s *r* value.

10.7717/peerj.6213/supp-5 Table S5 Selection of climatic variables and best overall model for annual tree height increment Preselected best seven transfer distance climatic variables for tree height (Step 1). Selection was based on the AIC and the significance (*P*) of the transfer distance climatic quadratic term. Preselected seed source climatic variables (Step 2). Selection was based on Spearman’s rank correlation coefficient (*r*), between population means across sites and the seed source climatic variable. The best full model (Step 3) were selected on the basis of AIC value (after fitting the model with individual values). In each step, models are sorted by their AIC or absolute Spearman’s *r* value.

10.7717/peerj.6213/supp-6 Table S6 Statistics of the mixed model analysis of annual tree height increment for the “overall” model (all the four species) Akaike Information Criterion (AIC), estimated parameters, contribution to total variance of random components and significance.

10.7717/peerj.6213/supp-7 Figure S1 Predicted tree height for the four species based on the best fitted model for (a) *Fagus sylvatica* and for (b) *Pinus sylvestris.* For F. sylvatica, the two selected climatic variables were: annual dryness index as transfer distance (D term of the mixed model), and summer mean minimum temperature as climate of the seed source (C term of the mixed model). For P. sylvestris, the two selected climatic variables were: annual dryness index as transfer distance (D term of the mixed model), and autumn (Sep. - Nov.) mean temperature (°C) as climate of the seed source (C term of the mixed model). After fitting the model for each of the two previous species, in each case the model then was refitted for the other three species and their respective fixed effect parameters used for estimate the climatic response function. Larger positive values on the x axis indicate transfer to drier sites and/or warmer sites; negative values indicate transfer to colder and/or wetter sites; a value of zero indicates transfer to a test site with a climate similar to that of the site of provenance. For illustrative purposes on this plots, we selected extreme populations for each specie along the environmental gradient.

10.7717/peerj.6213/supp-8 Figure S2 Annual tree height increment response to climatic transfer distance Predicted annual tree height growth (cm) of species averages (solid lines and symbols) and of extreme populations per species [with more continental or less continental temperature differentials (TD) as seed source climatic variable; broken lines] vs. climatic transfer distance, for *Fagus sylvatica, Quercus petraea*, *Pinus sylvestris* and *Picea abies*. Positive values on the *x* axis indicate transfer to drier and/or warmer sites; negative values signify transfer to cooler and/or moister sites; zero stands for a climate similar to that at the site of provenance (further explanation in text).

10.7717/peerj.6213/supp-9 Appendix S1 Description of the provenance trials and data sources

10.7717/peerj.6213/supp-10 Appendix S2 Model selection

10.7717/peerj.6213/supp-11 Appendix S3 References of Supplementary Information

10.7717/peerj.6213/supp-12 Supplemental Information 1 Tree height and climate of seed source and tests sites Data set including averaged tree height (cm) per block of each population at each tests site; climate values for seed source (_s), tests site (_t) and climate transfer distance (_d).

## Additional Information and Declarations

### Data Availability

The following information was supplied regarding data availability: Raw data are provided in the Supplemental Files.
