# Supplementary material for: Common garden comparisons confirm inherited differences in sensitivity to climate change between forest tree species
Source: PeerJ. 2019 Jan 15;7:e6213. doi: 10.7717/peerj.6213 (PMC6338101; doi:10.7717/peerj.6213)
Supplement: Table S1 — Codes and definitions of climate variables as in Table 1. [file peerj-07-6213-s001.docx]

Table S1. List and basic data of populations tested. Codes and definitions of climate variables as in Table 1.

| Sp* | Population | Country | LAT | LON | Elevation | MAT | MAP | ADI |
| --- | --- | --- | --- | --- | --- | --- | --- | --- |
| Fsy | Nižbor | CZE | 50 | 14 | 480 | 6.9 | 531 | 0.0768 |
| Fsy | Torup | SWE | 55.57 | 13.2 | 40 | 7.7 | 641 | 0.0637 |
| Fsy | Bilowo | POL | 54.33 | 18.17 | 250 | 5.7 | 633 | 0.0594 |
| Fsy | Soignes | BEL | 50.83 | 4.42 | 110 | 9.9 | 810 | 0.0567 |
| Fsy | Aarnink | NLD | 51.93 | 6.73 | 45 | 9.4 | 787 | 0.0565 |
| Fsy | Westfield | GBR | 57.4 | -2.75 | 10 | 8.2 | 839 | 0.0451 |
| Fsy | Urach | DEU | 48.47 | 9.45 | 760 | 7 | 943 | 0.0421 |
| Fsy | Horni Plana | CZE | 48.85 | 14 | 990 | 4.8 | 1114 | 0.0309 |
| Fsy | Plateaux | FRA | 46.8 | 5.83 | 600 | 8.6 | 1550 | 0.0281 |
| Pab | Dobříš | CZE | 49.78 | 14.2 | 420 | 7.6 | 566 | 0.0762 |
| Pab | Wismar | DEU | 53.83 | 11.33 | 100 | 7.8 | 625 | 0.067 |
| Pab | Verior Revier | EST | 58.08 | 27.42 | 30 | 4.5 | 592 | 0.0662 |
| Pab | Witebsk | BLR | 55.08 | 30.17 | 150 | 4.9 | 630 | 0.0642 |
| Pab | Moravská Třebová | CZE | 49.75 | 16.67 | 570 | 6.3 | 618 | 0.0641 |
| Pab | Przerwanki | POL | 54.17 | 21.95 | 150 | 6.3 | 669 | 0.0639 |
| Pab | Urjala Honkolan | FIN | 61.1 | 23.5 | 100 | 3.7 | 564 | 0.0638 |
| Pab | Zwingenberg | DEU | 49.73 | 8.62 | 500 | 7.6 | 664 | 0.0632 |
| Pab | Marginea | ROU | 47.75 | 25.75 | 670 | 6.4 | 666 | 0.0628 |
| Pab | Bükkszentkereszt | HUN | 48.07 | 20.63 | 615 | 7.3 | 696 | 0.0624 |
| Pab | Terepetsk | RUS | 56.5 | 32 | 200 | 3.7 | 618 | 0.0618 |
| Pab | Nové Hrady | CZE | 48.83 | 14.8 | 500 | 7.4 | 781 | 0.0542 |
| Pab | Borovec | BGR | 42.25 | 23.58 | 1300 | 6.2 | 713 | 0.053 |
| Pab | Cosna | ROU | 47.33 | 25.17 | 1025 | 5.1 | 720 | 0.0516 |
| Pab | Jasina | UKR | 48.25 | 24.33 | 700 | 6.5 | 804 | 0.0513 |
| Pab | Val di Fiemme | ITA | 46.28 | 11.47 | 1100 | 6.9 | 813 | 0.0503 |
| Pab | Bogstad | NOR | 59.97 | 10.65 | 160 | 5.1 | 776 | 0.0488 |
| Pab | Baden-Baden | DEU | 48.77 | 8.23 | 500 | 8.2 | 907 | 0.0478 |
| Pab | Kláštor pod Znievom | SVK | 48.97 | 18.87 | 700 | 5.8 | 831 | 0.0472 |
| Pab | Vallen | SWE | 62.33 | 14.13 | 450 | 2.3 | 671 | 0.0472 |
| Pab | Hrabušice | SVK | 48.98 | 20.18 | 900 | 4.9 | 770 | 0.047 |
| Pab | Oestlandsgarn | NOR | 60.5 | 11 | 275 | 3.6 | 745 | 0.0468 |
| Pab | Burghausen | DEU | 48.17 | 12.82 | 500 | 7.7 | 935 | 0.0462 |
| Pab | Stuebing | AUT | 47.33 | 15.6 | 640 | 6.7 | 981 | 0.0416 |
| Pab | Cucureasa | ROU | 47.33 | 25 | 1410 | 2.9 | 747 | 0.0414 |
| Pab | Knittelfeld | AUT | 47.2 | 14.82 | 1300 | 2.8 | 793 | 0.0389 |
| Pab | Vorau | AUT | 47.4 | 15.88 | 1200 | 3.4 | 862 | 0.0369 |
| Pab | Gurktal | AUT | 46.87 | 14.28 | 1300 | 3 | 888 | 0.0354 |
| Pab | Veitsch-Neuberg | AUT | 47.65 | 15.58 | 1000 | 4.6 | 980 | 0.0352 |
| Pab | Liptovský Hrádok | SVK | 48.93 | 19.85 | 1125 | 3.8 | 1120 | 0.0291 |
| Pab | Crespato | CHE | 46.52 | 8.63 | 1120 | 6.2 | 1486 | 0.0246 |
| Pab | Baindt | DEU | 47.5 | 9.83 | 600 | 7.7 | 1799 | 0.0237 |
| Pab | Mali Lom | HRV | 44.75 | 14.97 | 1150 | 6.6 | 1976 | 0.0196 |
| Pab | Tavejanne | CHE | 46.3 | 7.12 | 1600 | 3.6 | 1753 | 0.017 |
| Psy | Voronezh | RUS | 51.63 | 39.47 | 135 | 5.9 | 510 | 0.0898 |
| Psy | Kiev/Borispol | UKR | 50.17 | 32.17 | 117 | 7.6 | 544 | 0.0871 |
| Psy | Tambov/Sosnovska | RUS | 53.2 | 41.33 | 137 | 4.8 | 527 | 0.0833 |
| Psy | Ryazan/Solotcha | RUS | 54.67 | 39.75 | 95 | 4.4 | 525 | 0.0809 |
| Psy | Sumy/Svyessa | RUS | 52.02 | 34 | 175 | 5.5 | 580 | 0.0745 |
| Psy | Bryansk/Gavansk | RUS | 53 | 34 | 179 | 4.8 | 570 | 0.0726 |
| Psy | Moscow/Kurovskoye | RUS | 55.53 | 38.95 | 120 | 4.2 | 571 | 0.0725 |
| Psy | Gorkiy/Gorodets | RUS | 56.67 | 43.47 | 106 | 3.7 | 596 | 0.0691 |
| Psy | Elwa | EST | 58.17 | 26.47 | 85 | 4.5 | 595 | 0.0648 |
| Psy | Kostroma/Kostroma | RUS | 58 | 42 | 165 | 2.9 | 626 | 0.0623 |
| Psy | Jaunielgawa | LVA | 56.45 | 25.17 | 78 | 5.3 | 641 | 0.062 |
| Psy | Murmansk/Kandalaksha | RUS | 67 | 32.55 | 11 | -0.3 | 490 | 0.0597 |
| Psy | Karelya/Pryazha | RUS | 61.67 | 33.67 | 141 | 1.8 | 612 | 0.0555 |
| Psy | Arkhangelsk/Plyesetsk | RUS | 62.9 | 40.4 | 105 | 1.1 | 638 | 0.0529 |
| Qpe | Syców | POL | 51.18 | 17.93 | 210 | 8 | 577 | 0.076 |
| Qpe | Bercé | FRA | 47.81 | 0.39 | 157 | 10.5 | 712 | 0.0667 |
| Qpe | Vouillé | FRA | 46.6 | 0.18 | 130 | 11.2 | 745 | 0.0661 |
| Qpe | Luss | DEU | 52.83 | 10.32 | 110 | 8.2 | 692 | 0.0614 |
| Qpe | Dreuille | FRA | 46.46 | 2.89 | 270 | 10.5 | 778 | 0.0612 |
| Qpe | Bolu | TUR | 40.92 | 31.67 | 1200 | 8.6 | 721 | 0.0606 |
| Qpe | Dymock | GBR | 51.95 | -2.45 | 70 | 9.6 | 717 | 0.0602 |
| Qpe | Rantzau | DEU | 53.71 | 9.76 | 10 | 8.3 | 767 | 0.0554 |
| Qpe | Londal | DNK | 56.07 | 9.6 | 25 | 7.5 | 736 | 0.0547 |
| Qpe | Elmstein | DEU | 49.36 | 7.87 | 470 | 7.7 | 755 | 0.0543 |
| Qpe | Reckling | DEU | 51.77 | 7.17 | 75 | 9.4 | 818 | 0.0543 |
| Qpe | Blakeney | GBR | 51.79 | -2.49 | 91 | 9.5 | 804 | 0.0534 |
| Qpe | Bussière | FRA | 47.76 | 5.49 | 330 | 9.6 | 890 | 0.0515 |
| Qpe | Hald Ege | DNK | 56.13 | 9.4 | 250 | 6.2 | 770 | 0.0477 |

* Fsy = *Fagus sylvatica*. Pab = *Picea abies*. Psy = *Pinus sylvestris*. Qpe = *Quercus petraea*. MAT = Mean Annual Temperature. MAP = Mean Annual Precipitation. ADI = Annual Dryness Index.
