# Supplementary material for: Common garden comparisons confirm inherited differences in sensitivity to climate change between forest tree species
Source: PeerJ. 2019 Jan 15;7:e6213. doi: 10.7717/peerj.6213 (PMC6338101; doi:10.7717/peerj.6213)
Supplement: Table S2 — See Table 1 for codes and definition of climate variables. [file peerj-07-6213-s002.docx]

Table S2. Test sites per species. See Table 1 for codes and definition of climate variables.

| Sp* | Site | Country | LAT | LON | Elevation  m a.s.l. | MAT  °C | MAP  mm | ADI  index |
| --- | --- | --- | --- | --- | --- | --- | --- | --- |
| Fsy | Bucsuta | HUN | 46.59 | 16.86 | 220 | 10.9 | 770 | 0.0672 |
| Fsy | Hahnengrün | DEU | 49.94 | 11.80 | 789 | 6.8 | 812 | 0.0487 |
| Fsy | Harre | BEL | 50.37 | 5.67 | 480 | 8.8 | 1108 | 0.0389 |
| Fsy | Mláčik | SVK | 48.62 | 18.99 | 850 | 6 | 834 | 0.0481 |
| Fsy | Oosterend | NLD | 51.97 | 5.69 | 30 | 10.3 | 874 | 0.0539 |
| Fsy | Straža | SVN | 45.79 | 15.04 | 545 | 10 | 1354 | 0.0359 |
| Pab | Abild | SWE | 56.95 | 12.73 | 65 | 7.3 | 820 | 0.0495 |
| Pab | Lisjo | SWE | 59.72 | 16.08 | 65 | 5.6 | 637 | 0.0608 |
| Pab | Lappkojberget | SWE | 63.42 | 18.67 | 220 | 1.7 | 616 | 0.0533 |
| Pab | Krynica | POL | 49.47 | 21 | 728 | 5.3 | 867 | 0.0424 |
| Pab | Nyírjes | HUN | 47.89 | 19.95 | 565 | 7.6 | 666 | 0.0645 |
| Psy | Kharkov | UKR | 49 | 37.5 | 137 | 7.8 | 511 | 0.0936 |
| Psy | Kovrov | RUS | 57 | 42 | 129 | 4.3 | 645 | 0.0640 |
| Psy | Davidovka | RUS | 51 | 39 | 127 | 6.8 | 561 | 0.0830 |
| Psy | Egyházashetye | HUN | 47.17 | 17.12 | 134 | 10.5 | 589 | 0.0861 |
| Qpe | Sprakensehl | DEU | 52.77 | 10.48 | 115 | 9 | 667 | 0.0666 |
| Qpe | Walkenried | DEU | 51.601 | 10.66 | 301 | 8.5 | 709 | 0.0607 |
| Qpe | Eitorf | DEU | 50.77 | 7.18 | 100 | 10.4 | 740 | 0.0646 |
| Qpe | Eppenbrunn | DEU | 49.1 | 7.67 | 330 | 9.4 | 710 | 0.0644 |
| Qpe | Müncheberg | DEU | 52.5 | 14.05 | 70 | 9.5 | 564 | 0.0834 |
| Qpe | Plön | DEU | 54.1 | 10.23 | 40 | 8.7 | 738 | 0.0581 |
| Qpe | Valby Hegn | DNK | 56.05 | 12.22 | 37 | 8.4 | 592 | 0.0716 |
| Qpe | Gl-Estrup | DNK | 56.43 | 10.35 | 15 | 8.1 | 588 | 0.0693 |
| Qpe | Toftlund | DNK | 55.2 | 9.07 | 25 | 8.3 | 857 | 0.0480 |
| Qpe | Petite Charnie | FRA | 48.09 | -0.17 | 150 | 11.2 | 749 | 0.0659 |
| Qpe | Vierzon | FRA | 47.26 | 2.13 | 170 | 11.5 | 709 | 0.0717 |
| Qpe | Vincence | FRA | 46.97 | 3.63 | 235 | 11.4 | 928 | 0.0547 |
| Qpe | Sillegny | FRA | 48.99 | 6.13 | 201 | 10.6 | 719 | 0.0675 |
| Qpe | Wykeham | GBR | 54.26 | -0.54 | 130 | 9.6 | 807 | 0.0535 |
| Qpe | Waverley | GBR | 52.33 | -1.48 | 90 | 10 | 638 | 0.0708 |
| Qpe | Hogwood | GBR | 51.08 | -0.556 | 40 | 10.7 | 748 | 0.0634 |
| Qpe | Dean | GBR | 51.8 | -2.54 | 172 | 9.8 | 917 | 0.0481 |
| Qpe | Glencors | GBR | 55.85 | -3.22 | 193 | 8.4 | 925 | 0.0424 |
| Qpe | Kórnik | POL | 52.24 | 17.08 | 75 | 8.9 | 540 | 0.0845 |
| Qpe | Estanbul | TUR | 41.00 | 29.00 | 200 | 13.4 | 757 | 0.0749 |
| Qpe | Sakarya | TUR | 40.01 | 30.01 | 100 | 14.6 | 523 | 0.1159 |
| Qpe | Bolu | TUR | 40.01 | 32.00 | 1550 | 8.4 | 448 | 0.1013 |
| Qpe | Kastamonu | TUR | 41.01 | 33.01 | 1050 | 9.6 | 668 | 0.0714 |

* Fsy = *Fagus sylvatica*. Pab = *Picea abies*. Psy = *Pinus sylvestris*. Qpe = *Quercus petraea*.

MAT = Mean Annual Temperature. MAP = Mean Annual Precipitation. ADI = Annual Dryness Index.
