# Supplementary material for: Common garden comparisons confirm inherited differences in sensitivity to climate change between forest tree species
Source: PeerJ. 2019 Jan 15;7:e6213. doi: 10.7717/peerj.6213 (PMC6338101; doi:10.7717/peerj.6213)
Supplement: Table S3 — Age of measurements considered are indicated. [file peerj-07-6213-s003.docx]

Table S4. Time periods for estimated climate of seed sources and for climate at test sites. Age of measurements considered are indicated.

| Code | Species | Seed collection | Sowing | Planting date | Date of measurements | Age from planting (years) | Climate Period Seed Source | Climate Period Field test |
| --- | --- | --- | --- | --- | --- | --- | --- | --- |
| Fsy | *Fagus sylvatica* |  | 1996 | 1998 | 2006 | 8 | 1961-1990 | 1998-2006 |
| Pab | *Picea abies* | 1964-1966 |  | 1968 | 1984 | 16 to 18 | 1941-1970 | 1968-1994 |
| Psy | *Pinus sylvestris* | 1974 | 1974 (URSS)  1976 (HUN) | 1976 (URSS)  1978 (HUN) | 1988-1994 | 12 to 17 | 1941-1970 | 1976-1994 |
| Qpe | *Quercus petraea* | 1989 | 1990 | 1992 | 2000 | 8 | 1961-1990 | 1990-2000 |
