# Supplementary material for: Common garden comparisons confirm inherited differences in sensitivity to climate change between forest tree species
Source: PeerJ. 2019 Jan 15;7:e6213. doi: 10.7717/peerj.6213 (PMC6338101; doi:10.7717/peerj.6213)
Supplement: Table S4 — Preselected best seven transfer distance climatic variables for tree height (Step 1). Selection was based on the AIC and the significance (P) of the transfer distance climatic quadratic term. Preselected seed source climatic variables (Step 2). Selection was based on Spearman’s rank correlation coefficient (r), between population means across sites and the seed source climatic variable. The best full model (Step 3) were selected on the basis of AIC value (after fitting the model with individual values). In each step, models are sorted by their AIC or absolute Spearman’s r value. [file peerj-07-6213-s004.docx]

Table S5. Selection of climatic variables and best overall model for tree height. Preselected best seven transfer distance climatic variables for tree height (Step 1). Selection was based on the AIC and the significance (*P*) of the transfer distance climatic quadratic term. Preselected seed source climatic variables (Step 2). Selection was based on Spearman’s rank correlation coefficient (*r*), between population means across sites and the seed source climatic variable. The best full model (Step 3) were selected on the basis of AIC value (after fitting the model with individual values). In each step, models are sorted by their AIC or absolute Spearman’s *r* value.

| Climatic variable | Code | | AIC* | *P* of quadratic term |
| --- | --- | --- | --- | --- |
| Step 1: Selection of transfer distance (_td) |  | |  |  |
| Annual Dryness Index ((DD>5)^1/2^ / MAP) | ADI_td | | 22103.9 | 0.0841 |
| Summer (Jun. - Aug.) mean temperature (°C) | TAVE_sm_td | | 22104.6 | <0.0001 |
| Spring (Mar. - May) mean temperature (°C) | TAVE_sp_td | | 22107.0 | <0.0001 |
| Spring mean minimum temperature (°C) | TMIN_sp_td | | 22108.6 | <0.0001 |
| Spring mean maximum temperature (°C) | TMAX_sp_td | | 22116.7 | <0.0001 |
| Autumn mean maximum temperature (°C) | TMAX_at_td | | 22124.1 | <0.0001 |
| Mean annual temperature (°C) | MAT_td | | 22129.4 | <0.0005 |
|  |  | |  |  |
| Step 2: Selection of seed source (_ss) |  | Spearman’s *r* | | *P* of Spearman’s coefficient |
| Temperature difference between MWMT and MCMT or continentality (°C) | TD_ss | | 0.403 | <0.0001 |
| Mean coldest month temperature (°C) | MCMT_12_ss | | -0.354 | <0.0001 |
| Winter (Dec.(prev. yr) - Feb.) mean temperature (°C) | TAV_wt_ss | | -0.342 | <0.0001 |
| Winter mean maximum temperature (°C) | TMAX_wt_ss | | -0.342 | <0.0001 |
| Winter mean minimum temperature (°C) | TMIN_wt_ss | | -0.339 | <0.0001 |
| Degree-days below 0°C, chilling degree-days | DD_0_ss | | 0.338 | <0.0001 |
| Extreme minimum temperature over 30 years | EMT_ss | | -0.327 | <0.0001 |
|  |  | |  |  |
| Step 3: Selection of full model (best combination of climate transfer distance and climate at seed source) |  | | AIC |  |
| ADI_ td, TMAX_wt _ss |  | | 22089.6 |  |
| ADI _ td, TAV_wt _ss |  | | 22089.6 |  |
| ADI_ td, TD _ss |  | | 22089.7 |  |
| ADI _ td, TMIN_wt_ss |  | | 22089.8 |  |
| ADI_ td, MCMT_ss |  | | 22090.6 |  |
| TMIN_sp_td, MCMT_ss |  | | 22092.4 |  |
| ADI_ td, EMT _ss |  | | 22093.0 |  |

* Lower value of Akaike Information Criterion (AIC) means a better fit of the model.
