# Supplementary material for: Common garden comparisons confirm inherited differences in sensitivity to climate change between forest tree species
Source: PeerJ. 2019 Jan 15;7:e6213. doi: 10.7717/peerj.6213 (PMC6338101; doi:10.7717/peerj.6213)
Supplement: Table S5 — Preselected best seven transfer distance climatic variables for tree height (Step 1). Selection was based on the AIC and the significance (P) of the transfer distance climatic quadratic term. Preselected seed source climatic variables (Step 2). Selection was based on Spearman’s rank correlation coefficient (r), between population means across sites and the seed source climatic variable. The best full model (Step 3) were selected on the basis of AIC value (after fitting the model with individual values). In each step, models are sorted by their AIC or absolute Spearman’s r value. [file peerj-07-6213-s005.docx]

Table S5. Selection of climatic variables and best overall model for annual tree height increment. Preselected best seven transfer distance climatic variables for tree height (Step 1). Selection was based on the AIC and the significance (*P*) of the transfer distance climatic quadratic term. Preselected seed source climatic variables (Step 2). Selection was based on Spearman’s rank correlation coefficient (*r*), between population means across sites and the seed source climatic variable. The best full model (Step 3) were selected on the basis of AIC value (after fitting the model with individual values). In each step, models are sorted by their AIC or absolute Spearman’s *r* value.

| Climatic variable | Code | | AIC* | *P* of quadratic term |
| --- | --- | --- | --- | --- |
| Step 1: Selection of transfer distance (_td) |  | |  |  |
| Annual Dryness Index ((DD>5)^1/2^ / MAP) | ADI_td | | 13353.8 | 0.0733 |
| Spring (Mar. - May) mean temperature (°C) | TAVE_sp_td | | 13367.4 | <0.0001 |
| Spring mean minimum temperature (°C) | TMIN_sp_td | | 13369.0 | <0.0001 |
| Spring mean maximum temperature (°C) | TMAX_sp_td | | 13372.3 | 0.0004 |
| Autumn mean maximum temperature (°C) | TMAX_at_td | | 13379.0 | 0.0036 |
| Mean annual temperature (°C) | MAT_td | | 13380.6 | 0.0071 |
| Summer (Jun. - Aug.) mean temperature (°C) | TAVE_sm_td | | 13381.0 | 0.0712 |
|  |  | |  |  |
| Step 2: Selection of seed source (_ss) |  | Spearman’s *r* | | *P* of Spearman’s coefficient |
| Summer precipitation (mm) | PPT_sm_ss | | 0.357 | 0.0024 |
| Mean summer (May to Sept.) precipitation (mm) | MSP_ss | | 0.328 | 0.0055 |
| Summer heat/moisture index ((MWMT)/(MSP/1000)) | SHM_ss | | -0.263 | 0.0281 |
| Winter precipitation (mm) | PPT_wt_ss | | -0.162 | 0.1796 |
| Autumn precipitation (mm) | PPT_at_ss | | -0.151 | 0.2118 |
| The Julian date on which frost-free period begins | bFFP_ss | | 0.142 | 0.2424 |
| Temperature difference between MWMT and MCMT or continentality (°C) | TD_ss | | 0.141 | 0.2439 |
|  |  | |  |  |
| Step 3: Selection of full model (best combination of climate transfer distance and climate at seed source) |  | | AIC |  |
| ADI_ td, TD _ss |  | | 13349.4 |  |
| ADI _ td, SHM_ss |  | | 13350.6 |  |
| ADI_ td, bFFP_ss |  | | 13352.1 |  |
| ADI _ td, MSP_ss |  | | 13354.0 |  |
| ADI_ td, PPT_sm_ss |  | | 13354.9 |  |
| ADI_ td, PPT_wt_ss |  | | 13360.2 |  |
| ADI_ td, PPT_at_ss |  | | 13361.1 |  |

* Lower value of Akaike Information Criterion (AIC) means a better fit of the model.
