# Supplementary material for: Common garden comparisons confirm inherited differences in sensitivity to climate change between forest tree species
Source: PeerJ. 2019 Jan 15;7:e6213. doi: 10.7717/peerj.6213 (PMC6338101; doi:10.7717/peerj.6213)
Supplement: Table S6 — Akaike Information Criterion (AIC), estimated parameters, contribution to total variance of random components and significance. [file peerj-07-6213-s006.docx]

Table S6. Statistics of the mixed model analysis of annual tree height increment for the “overall” model (all the four species): Akaike Information Criterion (AIC), estimated parameters, contribution to total variance of random components and significance.

| Parameter / components of variation of tree height | | Statistics | | |
| --- | --- | --- | --- | --- |
| Fixed effects | Estimated parameter or variance value | | %^*^ | *P* |
| AIC | | 13349.4 |  |  |
| Intercept | | 31.7 |  | 0.0028 |
| Temperature difference between mean of warmest and coldest month temperature, or continentality (TD_s), of the seed source | | -0.009 |  | 0.9474 |
| Annual dryness index transfer distance (ADI_d) | | 64.5 |  | 0.5023 |
| Annual dryness index transfer distance quadratic (ADI_d)^2^ | | -1348 |  | 0.0419 |
| Temperature difference x Annual dryness index transfer distance | | -5.9 |  | 0.1875 |
| *Random effects* | |  |  |  |
| *Species* | | 0 | 0.0 | 1 |
| *Site* | | 181.9 | 82.3 | <0.0001 |
| *Population(Species)* | | 3.27 | 1.5 | 0.0032 |
| *Error* | | 35.9 | 16.2 |  |
|  | |  |  |  |

^*^Percent contribution to total variance (where 100 % is the sum of variance of all random terms).
