# Supplementary figures and images for: Common garden comparisons confirm inherited differences in sensitivity to climate change between forest tree species
Source: PeerJ. 2019 Jan 15;7:e6213. doi: 10.7717/peerj.6213 (PMC6338101; doi:10.7717/peerj.6213)

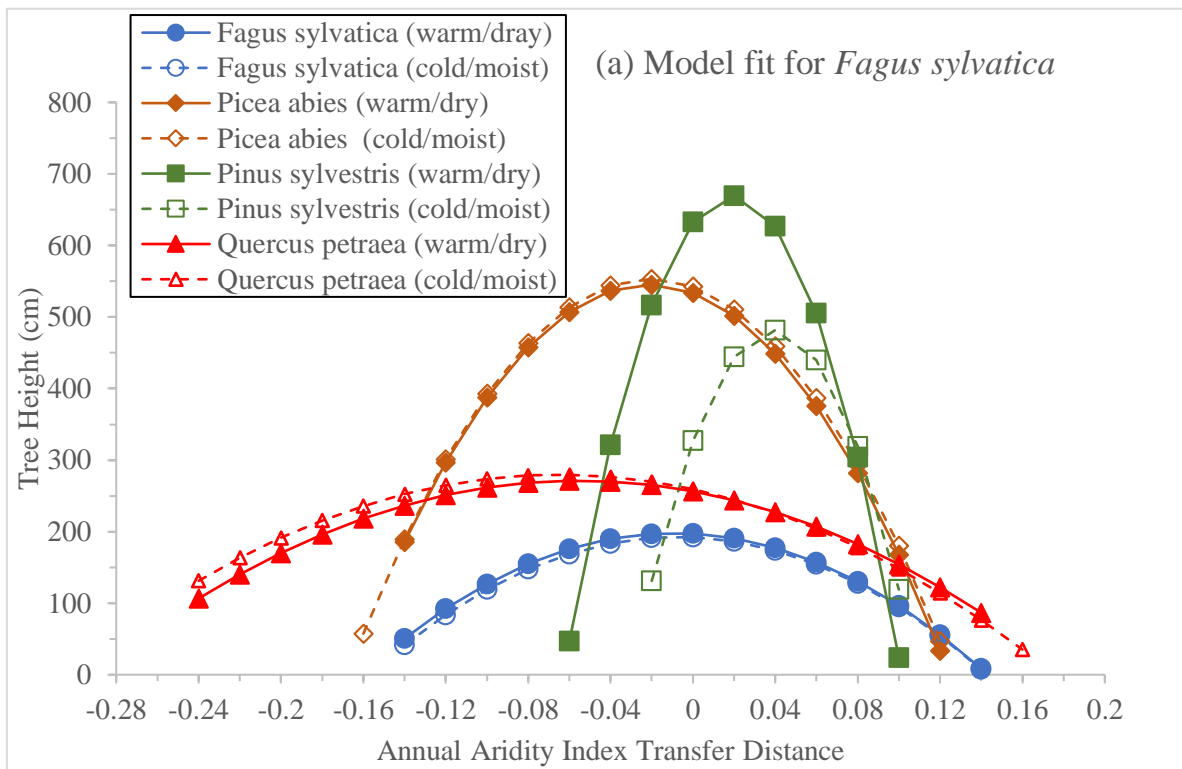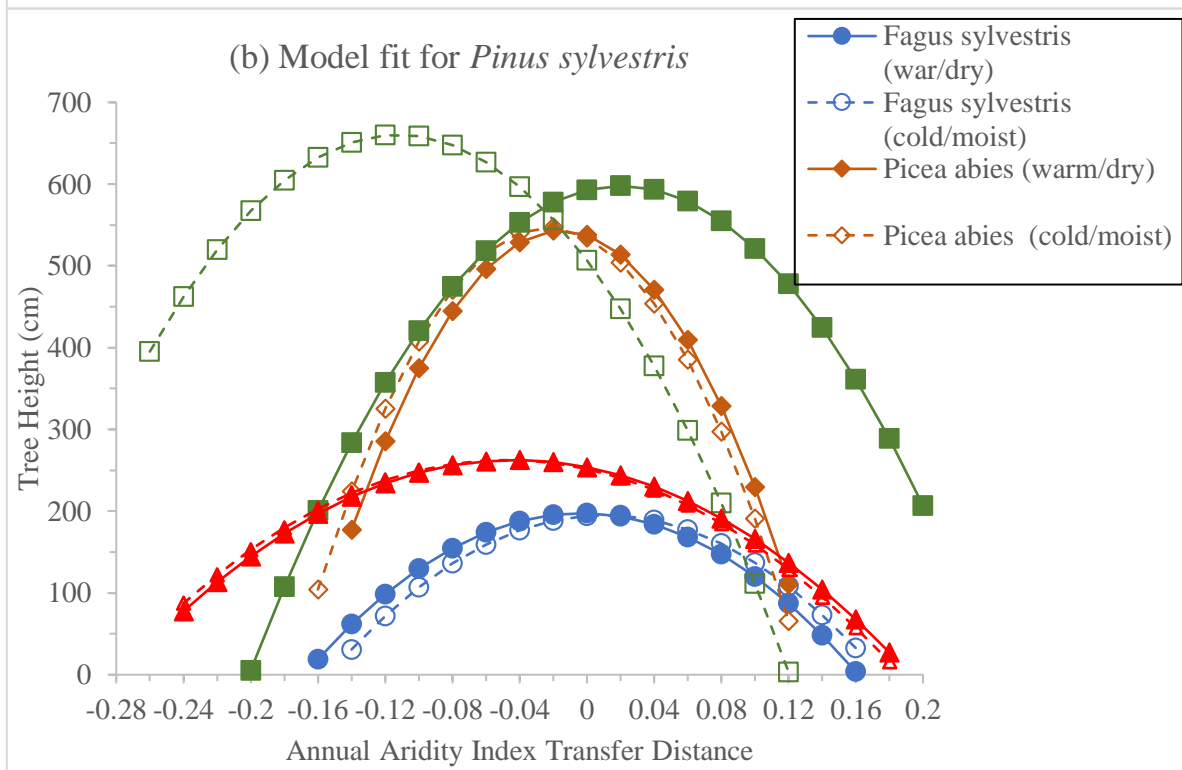

Supplement: Figure S1 — For F. sylvatica, the two selected climatic variables were: annual dryness index as transfer distance (D term of the mixed model), and summer mean minimum temperature as climate of the seed source (C term of the mixed model). For P. sylvestris, the two selected climatic variables were: annual dryness index as transfer distance (D term of the mixed model), and autumn (Sep. - Nov.) mean temperature (°C) as climate of the seed source (C term of the mixed model). After fitting the model for each of the two previous species, in each case the model then was refitted for the other three species and their respective fixed effect parameters used for estimate the climatic response function. Larger positive values on the x axis indicate transfer to drier sites and/or warmer sites; negative values indicate transfer to colder and/or wetter sites; a value of zero indicates transfer to a test site with a climate similar to that of the site of provenance. For illustrative purposes on this plots, we selected extreme populations for each specie along the environmental gradient. [file peerj-07-6213-s007.pdf]

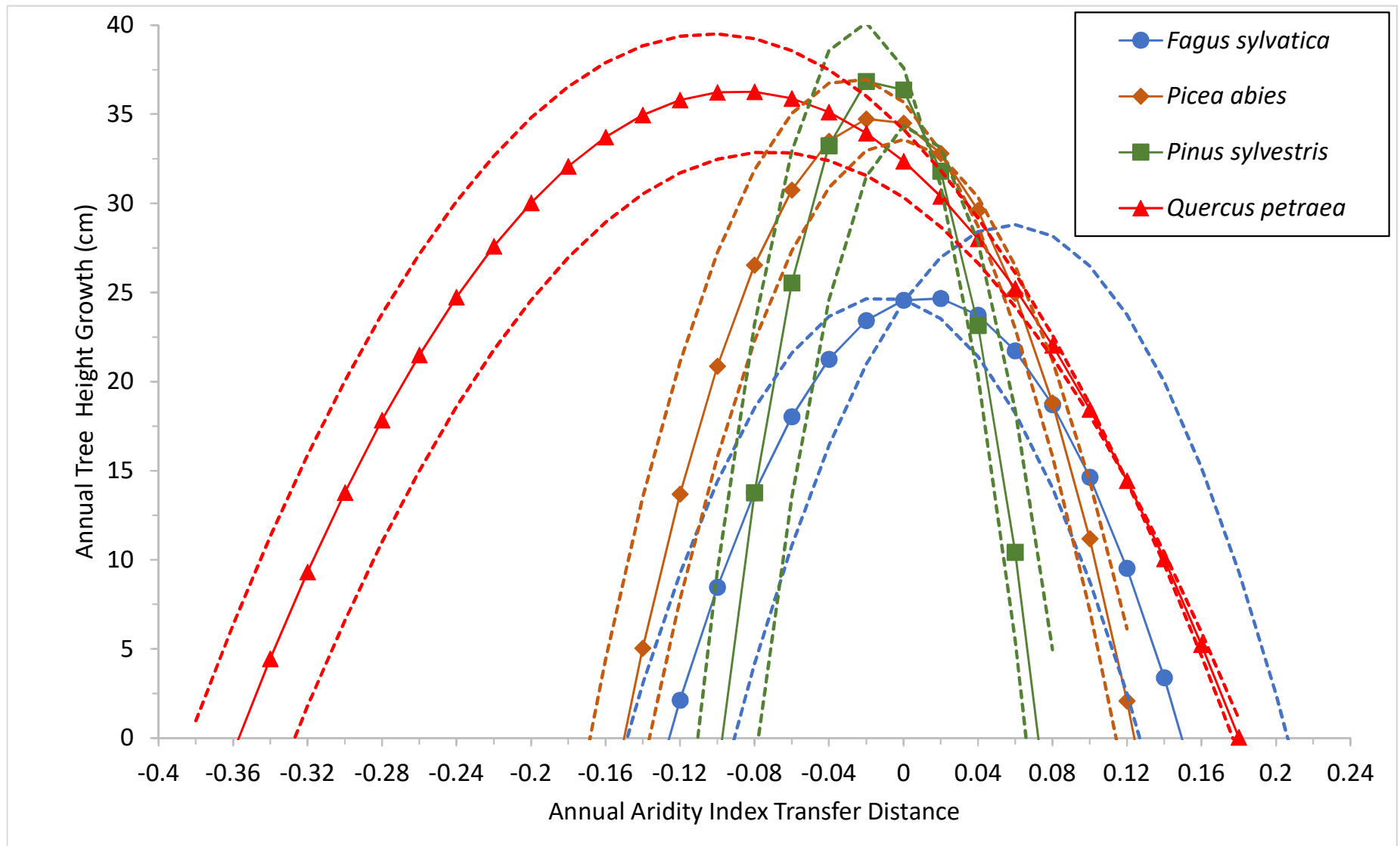

Supplement: Figure S2 — Predicted annual tree height growth (cm) of species averages (solid lines and symbols) and of extreme populations per species [with more continental or less continental temperature differentials (TD) as seed source climatic variable; broken lines] vs. climatic transfer distance, for Fagus sylvatica, Quercus petraea, Pinus sylvestris and Picea abies. Positive values on the x axis indicate transfer to drier and/or warmer sites; negative values signify transfer to cooler and/or moister sites; zero stands for a climate similar to that at the site of provenance (further explanation in text). [file peerj-07-6213-s008.pdf]
