# Supplementary material for: Common garden comparisons confirm inherited differences in sensitivity to climate change between forest tree species
Source: PeerJ. 2019 Jan 15;7:e6213. doi: 10.7717/peerj.6213 (PMC6338101; doi:10.7717/peerj.6213)
Supplement: Appendix S1 [file peerj-07-6213-s009.docx]

**APPENDIX 01.**

**Description of the provenance trials and data sources**

1. ***Picea abies***

**The international provenance test 1964/1968 of Norway spruce (IPTNS) initiated by IUFRO**

The detailed plans of the international trial were made by Klaus Stern and Wolfgang Langner (Institute of Forest Genetics and Forest Tree Breeding at Schmalenbeck, Germany). In spring 1964, 1300 samples were sown in the nurseries of the Institute.

Ultimately 1100 populations were available in necessary numbers at the end of the nursery period*.* This number was split up into 11 blocks of 100 populations. Each block can be regarded as a complete provenance trial in itself, containing material from the entire range of the collection. The 11 blocks were assumed to be equal in mean and within-block variance, thus directly comparable. The experimental unit of the trial is the block of 100 populations with 25 plants each, distributed in random single-tree plots. 20 trials were established in 13 countries, outplanted in spring 1968. The trial network was initially planned for an active observation period of 20 years.

The realization of the project was organized by the Forestry College of the Royal University at Stockholm under the guidance of Peter Krutzsch and carried out from the beginning under the auspices of the Provenance Trial Working Group, Section 22 of IUFRO.

The experimental background is described in the pubication KRUTZSCH, P. (1974). Detailed descriptive list and growth data of populations in various tests may be found in publications listed in Ujvári-Jármay et al. 2016.

The sources of data used for the present publication are listed in Table S7.

Table S7. *Picea abies* tests codes and data source

| *Test code, site and country* | *Data source* | *Remark* |
| --- | --- | --- |
| 6 Abild (SWE) | P. Krutzsch pers.comm. |  |
| 7 Lisjö (SWE) | P. Krutzsch pers.comm |  |
| 8 Lappkojberget (SWE) | P. Krutzsch pers.comm |  |
| 19 Krynica (POL) | Sabor 1997 |  |
| 20 Nyirjes (HUN) | Ujvári-Jármay et al. 2016 |  |

1. ***Pinus sylvestris***

**The VNIILM provenance test network of Scots pine**

Provenance variation of Scots pine has been well investigated in multiple series of national and international provenance trials; however, provenances from East Europe, but especially from the Asian part of the distribution range were insufficiently represented. In 1972, the (former) All-Soviet Research Institute of Forest Amelioration (Pushkino, RUS) initiated large-scale population sampling throughout the former Soviet Union. The original concept of the coordinator, Y. P. Prokazin, included the preservation of parts of the maternal stands and establishment of *in situ* progeny stands for control stands.

The seed samples were collected from 113 specific, presumably autochthonous, stands representing in Europe regions of 2° latitude and 4-5° longitude in the western and southern parts of the distribution area, 2-3° latitude and 6-7° longitude in the northern and north-eastern sector of the range and in the Asian part of the former Soviet Union regions of 5-6° latitude and 10-12° longitude. The seeds had been extracted locally and the coordinator distributed the seed lots among the participating institutes.

The deployment of the 37 trials took place between 1975 and 1978; four of them had been perished during the establishment phase. The experimental layout was not completely uniform; most trials include 3 replicates (blocks) with randomised provenance plots within replicates. The number of included provenances ranges from 10 to 90, averaging 40. The size of the plots is also very variable, depending on the availability of the seedlings per provenance.

Initiated by C. Mátyás (Forest Research Institute, Hungary), four additional trials of this series had been established outside the Soviet Union, in Hungary. These experiments include in addition to the Soviet set, population samples from Hungary, Poland, Turkey and the former Czechoslovakia, German Democratic Republic and Yugoslavia (Mátyás 1987).

Detailed description on the experimental background and summarized results of height growth measurements and survival data have been published after two decades for a large number of ex-Soviet test locations by Shutyaev and Giertych (1997). Detailed maps of sampled locations and trials, as well as statistics of growth performance of provenances were published by Shutyaev and Giertych in 2000. Test sites included in the present work are as in Table S8.

Table S8. *Pinus sylvestris* test site codes, data sources and ages at measurement (after field planting).

| Test code, site and country | Data source | Age after planting (years) |
| --- | --- | --- |
| 13 Izyum (UKR) | Patlaí and Zhurova 1991* | 15 |
| 16 Kovrov (RUS) | Malkin and Osipov 1990* | 15 |
| 17 Davidovka (RUS) | Shutyaev and Dyemidyenko 1995* | 17 |
| 1002 Egyházashetye (HUN) | Unpublished, L Nagy, Forest Research Institute, HUN | 16 |

*Original sources reviewed and summarized by Shutyaev and Giertych (1997).

1. ***Fagus sylvatica***

**The International Beech Provenance Trials of 1993/95 and 1996/98 in Europe**

In 1995 and 1998 international beech provenance trials were established in Europe, organized by the Institute for Forest Genetics, Grosshandorf, Germany, initiated by H.J. MUHS and G. VON WÜHLISCH (VON WÜHLISCH 2007). With support of a large number of participants, and the coordination of the Von-Thuenen Institute, among other institutions, 42 tests were successively planted across Europe.

The main objective of these experiments was to identify intraspecific variation in adaptive traits in order to be able to determine the most suitable population at a given site, and to make predictions for future distribution range of beech under changing environmental conditions.

Seeds were collected from the whole distribution area and raised in Hamburg, in a nursery until age two. After two years they were planted out to the trial sites. The layout of the planting was the same at each site. Provenances were planted in randomized plots with 10 m x 10 m plot size, 50 plants per plot (5 rows each with 10 plants) and replicated in three blocks across the site.

An important support for international cooperation and creation of a central dataset was initiated by the European COST E52 cooperative action which provided a platform for the participants of the experimental series to share experiences and measurement results. The main data and conclusions of the COST action have been published in Spain by ALIA ET AL. (2011).

Table S9. *Fagus sylvatica* tests codes, data sources and ages at measurement (after field planting).

| Test code, site and country | Data source | Age after planting (years) |
| --- | --- | --- |
| 15 Bucsuta HUN | COST Action E52, beech database | 8 |
| 23 Hahnengruen GER | COST Action E52, beech database | 8 |
| 06 Harre BEL | COST Action E52, beech database | 8 |
| 20 Mlacik SVK | COST Action E52, beech database | 8 |
| 08 Oosterens NED | COST Action E52, beech database | 8 |
| 12 Straza SVN | COST Action E52, beech database | 8 |

1. ***Quercus petraea***

Information from Supplementary information of Saénz-Romero et al (2017).

*Seed sources*

It was established an extensive collection of seeds from natural populations across the entire distribution range of *Q. petraea* between 1986 and 1992, with the aim of carrying out multisite provenance tests. As acorns cannot be stored for more than one year, and the seed crop is usually unevenly distributed, collections were made over several years, to ensure a thorough sampling of the species distribution. As flowering and fruiting were irregular, visits for collection were made over successive years for several populations. If the final harvest from these populations was too small, they were discarded. Each seed lot consisted of a bulk of ground-harvested acorns from collection points systematically distributed over an area of 5 to 40 ha, depending on tree density. We harvested between 10 kg and 100 kg of seed from each collection area. The seed lots were subsequently shared between the various partners in this project and used to raise seedlings independently, at the participating nurseries in each country.

The populations included in this analysis for *Quercus petraea* are the ones belonging to the so called “Madsen collection” (Tables S1; Madsen, 1990). Dr. Søren F. Madsen, a geneticist at the Danish Forest and Landscape Research Institute, initiated in 1989 a rangewide collection of a limited number of populations to be planted at a large number of test sites. Seedlings were obtained from these seeds and planted at 27 test sites (Fober, 1994; Ducousso et al 1996; Dağdaş & Gökdemir, 2002). A full documentation of the provenances is available from the Quercus Portal, a publicly accessible repository of oak genetic and genomic data (http://w3.pierroton.inra.fr:8006/materials). All the data related to this publication are stored at this site, together with all the metadata required.

*Field tests*

Unless otherwise stated, seedlings were planted out in the 27 field tests after two or three years in the nursery. Data were successfully recorded over a number of years for 23 field tests. The remaining four test sites were not included in the analysis due to problems with transplantation or insufficient data recording (Figure 1, Table S2). The French field tests covered a very large set of populations for which it was not possible to collect seed over the entire distribution range during a single year, due to the uneven nature of seed production. Transplantations were thus carried out over four years (1990, 1991, 1993 and 1996) corresponding to four large sections (referred to as “tranches” in French). The Madsen collection populations were all planted in the spring of 1993 in the same “tranche” at the four French sites.

A randomized complete block design was adopted at each field site. At the French test sites, each complete block was further subdivided into nested incomplete micro-blocks, to account for the larger number of populations tested.

*Data collection and input for analysis.*

It was planned to establish and manage the multisite field tests synchronously, but logistical issues and funding constraints resulted in measurements being taken at different time points at different sites and differences in the number of variables measured between sites. We focused our analysis on height growth. These two attributes were repeatedly assessed, but at different time intervals, in the various plantations. For each individual tree, both traits were re-estimated 10 years after seed collection (eight years after planting), from the maximum amount of available information (see details below). Total height was assessed at different ages at the various test sites: 2, 5, 7, 8, 11, 15 and 20 years since plantation. For all the test sites considered, the 10 years after the seed collection time point was either very close to the real measurement time or was between two successive measurements. We standardized tree height at 10 years by fitting a linear model to each of the individual trees, based on all the available data.
