# Supplementary material for: Common garden comparisons confirm inherited differences in sensitivity to climate change between forest tree species
Source: PeerJ. 2019 Jan 15;7:e6213. doi: 10.7717/peerj.6213 (PMC6338101; doi:10.7717/peerj.6213)
Supplement: Appendix S2 [file peerj-07-6213-s010.docx]

**APPENDIX 02**

**Model selection**

Selection based on the methodology of Sáenz-Romero et al (2017), which in turn was based on Leites *et al*. (2012a, 2012b).

**Model selection for tree height**

*Selection of climatic variables for the full model*

The selection of the climatic variables that best explained the effect of climatic transfer distance (D term, model 1) and climate at the provenance (C term, model 1 in Main Text) on population growth, followed a three step procedure, as described by Leites *et al*. (2012a, 2012b).

1. STEP 1. For each climatic variable, we ran a reduced model including the climatic transfer distance fixed term (D), its quadratic term, and all the random terms, but excluding the fixed term relating to climate at the seed source. We then preselected all the models with a negative and significant or nearly significant (*P* ≤ 0.09) climatic distance quadratic term (β_2_ negative). We retained the seven best models, identified on the basis of the Akaike Information Criterion, AIC (Akaike, 1973). In this step, we aimed to include the transfer distance climatic variables consistent with observations made in other tree species that, in general, tree height growth curves follow a quadratic shape, with the highest (optimal) value when the transfer distance is close to zero, and a decrease in performance when the population is moved to sites with a very different climate, regardless of the direction of change, to a much colder or warmer habitat or to a much drier or wetter site (Leites *et al.,* 2012a, 2012b*;* Rehfeldt *et al*., 2003).
2. STEP 2. We then selected the best candidate variables for climate at seed source (C), through the estimation of Spearman’s rank correlation coefficients (r) between population means (across sites) and climatic variables at the provenance site. We retained the best seven models identified on the basis of their Spearman’s rank correlation coefficients and the significance of the model (*P* ≤ 0.001) (Leites *et al*., 2012a). The aim of this step was to include climatic variables associated with genetic clines often responsible for molding genetic differentiation between populations in the model (Alberto *et al*., 2011; 2013).
3. STEP 3. We then ran 7 x 7 = 49 competing full models (model 1), each combining a pair (one preselected climatic variable for transfer distance and one for the climate at seed source) of the best climatic variables from the two previous model selection steps. The best model was selected on the basis of the AIC and the significance of the fixed terms. Finally, a series of plots was used to predict population means across a large series of hypothetical climatic transfer distance values, using the fixed terms of the models, to check that the quadratic response functions made biological sense, and to prevent the selection of a model with a low AIC value but no biological meaning.

*Results of three-step model selection*

The first step in the model selection process yielded the seven best models (lowest AIC values) with significant or nearly significant, negative quadratic terms of the climatic transfer distance variables for the following variables (in ascending order of AIC): Annual Dryness Index [ADI = (DD>5)^1/2^ / MAP], Summer (Jun. - Aug.) mean temperature (TAVE_sm), Spring (Mar. - May) mean temperature (TAV_sp), Spring mean maximum temperature (TMAX_sp), Spring mean minimum temperature (TMIN_sp), Autumn mean maximum temperature (TMAX_at), and Mean annual temperature (MAT) (Table S4).

The variable ADI indicates the relevance of the balance between daily temperatures above 5 °C (the one useful to plants to growth) and available moisture. Three of the seven preselected climatic variables are related to Spring temperature, indicating the relevance of temperature when the growth starts after the winter arrest.

Step two yielded the best seven models in terms of highly significant (P ≤ 0.0001) Spearman’s rank correlations between population means across sites and seed source climatic variables, for the following variables (in descending order of *r* values): Temperature difference between MWMT and MCMT or continentality (TD), Mean coldest month temperature (MTCM), Winter (Dec.(prev. yr) - Feb.) mean temperature (TAV_wt), Winter mean maximum temperature (TMAX_wt), Winter mean minimum temperature (TMIN_wt), Degree-days below 0°C, chilling degree-days (DD_0), Extreme minimum temperature over 30 years (EMT) (Table S4). Notice that six of the seven variables are directly related to cold winter temperatures, and of them, five variables had a negative correlation; that indicates that growth potential is limited by winter cold temperatures at the seed source.

Step three indicates that Annual dryness index (ADI) was, by far, the most relevant fixed term for climatic transfer distance during the final step of model selection, with **six** of the first best seven competing models from the 49 full models having ADI as a transfer distance variable.

The selected model, with ADI as the climatic transfer distance variable and Winter mean maximum temperature (TMAX_wt; °C) as the seed source climatic term, was one of two models with the lowest AIC value (22089.6) among the 49 competing models (Table S4), and with the most significant fixed terms (Table 1). The other model with the coincidentally the same AIC value was also with ADI as transfer distance although with winter average temperature (TAVE_wt; °C) as the seed source climatic term. However, these second model had their fixed terms less significant than the first model. Because that, we selected the model with ADI as the climatic transfer distance variable and Winter mean maximum temperature (TMAX_wt; °C) as the seed source climatic term.

**Model selection for annual tree height growth**

***Selection of climatic variables for the full model***

The selection of the climatic variables for annual tree height growth was the same as described above for tree height. We describe only minor changes as follows.

1. STEP 1. We preselected all the models with a negative and significant or nearly significant (*P* ≤ 0.08) climatic distance quadratic term (β_2_ negative). We retained the seven best models, identified on the basis of the Akaike Information Criterion, AIC (Akaike, 1973).
2. STEP 2. We selected the best seven candidate variables for climate at seed source (C), through the estimation of Spearman’s rank correlation coefficients (r) between population means (across sites) and climatic variables at the provenance site. Because only three climatic variables had significant (*P* ≤ 0.05) Spearman’s rank correlation coefficients, we included four additional climatic variables based on their highest correlation coefficients (r).
3. STEP 3. We then ran 7 x 7 = 49 competing full models (model 1), each combining a pair (one preselected climatic variable for transfer distance and one for the climate at seed source) of the best climatic variables from the two previous model selection steps. The best model was selected on the basis of the AIC. Finally, a series of plots was used to predict population means across a large series of hypothetical climatic transfer distance values, using the fixed terms of the models, to check that the quadratic response functions made biological sense, and to prevent the selection of a model with a low AIC value but no biological meaning.

***Results of three-step model selection***

The first step in the model selection process yielded the seven best models (lowest AIC values) with significant or nearly significant, negative quadratic terms of the climatic transfer distance variables for the following variables (in ascending order of AIC): Annual Dryness Index [ADI = (DD>5)^1/2^ / MAP], Spring (Mar. - May) mean temperature (°C; TAVE_sp), Spring mean minimum temperature (TMIN_sp), Spring mean maximum temperature (TMAX_sp), Autumn mean maximum temperature (TMAX_at), Mean annual temperature (MAT) and Summer (Jun. - Aug.) mean temperature (TAVE_sm) (Table S5).

Notice that all those seven preselected climatic variables are exactly the same than the seven transfer distance variables preselected for tree height, only shifting a little the order based on the AIC value of the model. AIC was again the one with the best AIC value.

Step two yielded the following seven preselected variables (in descending order of absolute *r* values): Summer precipitation (mm; PPT_sm), Mean summer (May to Sept.) precipitation (mm; MSP), Summer heat/moisture index (SHM = MWMT/(MSP/1000)), Winter precipitation (mm; PPT_wt), Autumn precipitation (mm; PPT_at), The Julian date on which frost-free period begins (bFFP), and Temperature difference between MWMT and MCMT or continentality (TD) (Table S5). Notice that four of the seven variables are directly related to precipitation; other two are related to temperatures, and one to the balance between temperature and precipitation during summer (SHM).

Step three indicates that Annual dryness index (ADI) was, by far, the most relevant fixed term for climatic transfer distance during the final step of model selection (similar as what happen for tree height analysis), where all the seven preselected models from the 49 full competing models had ADI as a transfer distance variable (Table S5). The selected model, based on its lowest AIC value (13349.4), had temperature difference between MWMT and MCMT or continentality (TD; °C) as the seed source climatic term. Notice that this best selected model contains the same climatic variables than the third best model for tree height (Table S4).
