# Supplementary material for: Common garden comparisons confirm inherited differences in sensitivity to climate change between forest tree species
Source: PeerJ. 2019 Jan 15;7:e6213. doi: 10.7717/peerj.6213 (PMC6338101; doi:10.7717/peerj.6213)
Supplement: Appendix S3 [file peerj-07-6213-s011.docx]

**Appendix 3.**

**References**

Akaike H (1973) Information theory and an extension of the maximum likelihood principle. *Proceedings of the Second International Symposium on Information Theory*, pp. 267–281.

Alberto F, Bouffier L, Louvet J-M *et al.* (2011) Adaptive responses for seed and leaf phenology in natural populations of sessile oak along an altitudinal gradient. *Journal of Evolutionary Biology,* 24, 1442–1454.

Alia R, Bozic G., Gömöry D., Huber G., Rasztovits E, von Wühlisch G. (2011) The survival and performance of beech provenances over a Europe-wide gradient of climate. pp. 115-126. in: Genetic Resources of European Beech (*Fagus sylvatica* L.) for Sustainable Forestry. Proceedings of the COST E52 Final Meeting. Monografías INIA, Serie Forestal, vol. 22.

Dağdaş S, Gökdemir G (2002) Istanbul-Fener Deneme Alanında Kurulu Sapsız Meşe (*Quercus petraea* Mattushka Liebl.) Orijin Denemesinin İlk Sekiz Yıllık Sonuçları. II. Ulusal Karadeniz Ormancılık Kongresi, 15-18 May 2002, II, pp. 431-437, Turkey.

Ducousso A, Guyon JP, Kremer A (1996) Latitudinal and altitudinal variation of bud burst in western populations of sessile oak (*Quercus petraea* (Matt.) Liebl.). *Annales des Sciences Forestiéres,* 53, 775–782

Fober H (1994) International provenance experiment with Quercus petraea (Matt.) Liebl. *Arboretum Kórnickie* 39, 109-124.

Krutzsch P. (1974) The IUFRO 1964/68 provenance test with Norway spruce (*Picea abies* (L.) Karst.). *Silvae Genetica*, 23, (1–3): 58–62.

Leites L P, Rehfeldt G E, Robinson A P, Crookston N L, Jaquish BC (2012a) Possibilities and limitations of using historic provenance tests to infer forest species growth responses to climate change. *Natural Resources Modeling,* 25, 409-433 .

Leites L P, Robinson AP, Rehfeldt GE, Marshall JD, Crookston NL (2012b) Height growth response to climatic changes differs among populations of Douglas-fir: a novel analysis of historic data. *Ecological Applications,* 22, 154 – 165.

Madsen SF (1990) International Provenance Trial with *Quercus petraea*. The 1989-Series of provenance experiments with *Quercus petraea* (Matt.) Liebl. Description of Seed Stands and Seed Collection. Technical report of Danish Forest Experiment Station, Denmark.

Malkin V K, Osipov A A. 1990. Razrabotat’ i vnyedrit’ myetody i tyekhnologii syelyektsii osnovnykh lyesoobrazuyushchikh porod s tsyel’yu poluchyeniya khozyaístvyenno-tsyennykh form i sortov. Final report on a research project. Ivantyeyevka, Moskovskaya Obl. Scientific Production Institute “Funduk” (VNIILM, NPO “Funduk”), 100 p.

Mátyás, C. 1987. Adaptációs folyamatok erdei fák populációiban. [Adaptation processes in forest tree populations.] Dissertation of DSc. Degree. Hungarian Academy of Sciences, Budapest, 173 p. + 48 p data tables (in Hungarian).

Patlai I N, Zhurova P T. 1991. Osushchyestvit’ ryealizatsiyu programmy rasshiryeniya gosudarstvyennoí syeti gyeografichyeskikh kul’tur, zakladky syeti populyatsionnoekologichyeskikh kultur luchshikh klimatipov, sovyermyenstvovat’ lyesosyemyennoye raíonirovaniye osnovnykh lyesoobrazuyushchikh porod. Final report on a research project. Khar’kov, Ukrainian Research Institute of Forestry (UkrNIILKhA), 115 p.

Rehfeldt, Gerald L. 2006. A spline model of climate for the Western United States. Gen. Tech. Rep. RMRS-GTR-165. Fort Collins, CO: U.S. Department of Agriculture, Forest Service, Rocky Mountain Research Station. 21 p.

Rehfeldt GE, Tchebakova NM, Milyutin LI, Parfenova YI, Wykoff WR, Kouzmina NA, *et al*. (2003) Assessing population responses to climate in *Pinus sylvestris* and *Larix* spp. of Eurasia with climate-transfer models. *Eurasian Journal of Forest Research,* 6, 83–98.

Sáenz-Romero C, Lamy JB, Ducousso A, Musch B, Ehrenmann F, Delzon S, Cavers S, Chałupka W, Dağdaş S, Hansen JK, Lee SJ, Liesebach M, Rau HM, Psomas A, Schneck V, Steiner W, Zimmermann NE, Kremer A (2017) Adaptive and plastic responses of *Quercus petraea* populations to climate across Europe. *Global Change Biology*. 23(7):2831–2847

Sabor J. (1997): *Picea abies* (L.) Karst. provenances and breeding. In: IUFRO Norway Spruce Symposium, Norway spruce breeding and genetic resources, Stara Lesna.

Shutyaev A M, Dyemidyenko V P. 1995. Analiz ranyeye zalozhyennykh gyeografichyeskikh kul’tur osnovnykh lyesoobrazuyushchikh porod. Final report on a research project, Voronyezh, Research Institute of Forest Genetics and Breeding (NIILGiS), 168 p.

Shutyaev A. M., Giertych M. 1997. Height growth variation in a comprehensive Euroasian provenance experiment of Pinus sylvestris L. Silvae Genetica 46:332–349.

Shutyaev, A. M., Giertych, M. 2000. Genetic subdivisions of the range of Scots pine (*Pinus sylvestris* L.) based on a transcontinental provenance experiment. *Silvae Genetica* 49(3):137-151

Újvári-Jármay É, NAGY L, MÁTYÁS C. 2016. The IUFRO 1964/68 Inventory Provenance Trial of Norway Spruce in Nyírjes, Hungary – results and conclusions of five decades**.** Acta Silvatica et Lignaria Hungarica 12, special issue, 178 p. DOI: 10.1515/aslh-2016-0001

von Wühlisch G. 2007. Series of international provenance trials of European beech. In: Improvement and Silviculture of Beech. Proc 7th Intern Beech Symp. IUFRO Res Gr 1.10.00, RIFR, Teheran, Iran. pp. 135-144.

Wang T, Hamann A. 2012*.* ClimateEU v4.63, a program to generate climate annual, seasonal and monthly data for Europe. Centre for Forest Conservation Genetics, Department of Forest Sciences, University of British Columbia, and Department of Renewable Resources, University of Alberta, Canada. 6 p.
